# Identification of Potential SARS-CoV-2 Main Protease and Spike Protein Inhibitors from the Genus *Aloe*: An In Silico Study for Drug Development

**DOI:** 10.3390/molecules26061767

**Published:** 2021-03-21

**Authors:** Mohamed E. Abouelela, Hamdy K. Assaf, Reda A. Abdelhamid, Ehab S. Elkhyat, Ahmed M. Sayed, Tomasz Oszako, Lassaad Belbahri, Ahmed E. El Zowalaty, Mohamed Salaheldin A. Abdelkader

**Affiliations:** 1 Department of Pharmacognosy, Faculty of Pharmacy, Al-Azhar University, Assiut-Branch, Assiut 71524, Egypt; m_abouelela@azhar.edu.eg (M.E.A.); hamdyss200@azhar.edu.eg (H.K.A.); reda.ahmed@azhar.edu.eg (R.A.A.); elkhayat@azhar.edu.eg (E.S.E.); 2 Department of Pharmacognosy, Faculty of Pharmacy, Nahda University, Beni-Suef 62513, Egypt; Ahmed.mohamed.sayed@nub.edu.eg; 3 Department of Forest Protection, Forest Research Institute, 05-090 Sekocin Stary, Poland; T.Oszako@ibles.waw.pl; 4 Laboratory of Soil Biology, University of Neuchatel, 2000 Neuchatel, Switzerland; 5 Sahlgrenska Center for Cancer Research, Department of Surgery, Institute of Clinical Sciences, University of Gothenburg, 405 30 Gothenburg, Sweden; 6 Wallenberg Centre for Molecular and Translational Medicine, University of Gothenburg, 405 30 Gothenburg, Sweden; 7 Department of Pharmacognosy, Faculty of Pharmacy, Sohag University, Nasr City, Sohag 82524, Egypt

**Keywords:** COVID-19, SARS-CoV-2, *Aloe*, docking, MD simulation, main protease, spike glycoprotein

## Abstract

Severe acute respiratory syndrome coronavirus (SARS-CoV-2) disease is a global rapidly spreading virus showing very high rates of complications and mortality. Till now, there is no effective specific treatment for the disease. *Aloe* is a rich source of isolated phytoconstituents that have an enormous range of biological activities. Since there are no available experimental techniques to examine these compounds for antiviral activity against SARS-CoV-2, we employed an in silico approach involving molecular docking, dynamics simulation, and binding free energy calculation using SARS-CoV-2 essential proteins as main protease and spike protein to identify lead compounds from *Aloe* that may help in novel drug discovery. Results retrieved from docking and molecular dynamics simulation suggested a number of promising inhibitors from *Aloe*. Root mean square deviation (RMSD) and root mean square fluctuation (RMSF) calculations indicated that compounds **132, 134**, and **159** were the best scoring compounds against main protease, while compounds **115, 120**, and **131** were the best scoring ones against spike glycoprotein. Compounds **120** and **131** were able to achieve significant stability and binding free energies during molecular dynamics simulation. In addition, the highest scoring compounds were investigated for their pharmacokinetic properties and drug-likeness. The *Aloe* compounds are promising active phytoconstituents for drug development for SARS-CoV-2.

## 1. Introduction

SARS-CoV-2, a novel coronavirus disease caused by Severe Acute Respiratory Syndrome Coronavirus 2, an RNA β-coronavirus, poses an increasing threat to human health. To date, SARS-CoV-2 has infected 105 million people worldwide (https://www.worldometers.info/, accessed on 20 February 2021). The disease is manifested by fever, cough, dyspnea and pneumonia with unknown etiology that worsens over time and can lead to death [1,2]. In addition, SARS-CoV-2 patients develop low levels of neutralizing antibodies leading to prolonged disease [3]. Entry of SARS-CoV-2 into host cells is a critical factor in its pathogenesis. The surface-anchored spike proteins of SARS-CoV-2 are key determinants of viral entry. They bind to surface receptors on host cells, then the virus enters endosomal pathway followed by fusion of viral and lysosomal membranes [4,5]. SARS-CoV-2 spike protein has N-terminus S1 domain, a receptor binding domain (RBD) that recognizes and binds angiotensin-converting enzyme 2 (ACE2) [6,7,8]. Proteolytic activation of SARS-CoV-2 spike proteins is mediated by the cell surface protease TMPRSS2, a process crucial for membrane fusion and viral entry [9]. Recent studies have shown that viral entry depends on the component of the head spike that recognizes the ACE2 receptor. In addition, structural and energetic analysis have shown that high-frequency contacts between ACE2 and SARS-CoV-2 spike protein lead to local conformational stability and large energetic cost was required for virus-cell collision at early stage facilitating cell entry [8,10,11].Coronavirus main protease (M^pro^, also known as 3CL^pro^), is the best characterized drug target, with no known human protease having the same cleavage pattern, so its inhibition leads to specific blockade of viral replication [12]. Spike glycoprotein (S protein) and its RBD are important targets for therapeutic intervention that target host cell recognition and the membrane fusion process [13]. As a global health emergency, abundant collaborative efforts have rapidly emerged to investigate the effectiveness of different therapies as antiviral, monoclonal antibodies, immune-therapies, and vaccines [14]. Current antiviral therapies for other viruses as SARS-CoV-1, MERS-CoV and HIV as well as antimalarial drugs have been inspected for their activity against SARS-CoV-2. For instance, the antimalarial drug hydroxychloroquine blocks viral cell entry by inhibiting glycosylation of host receptors and proteolytic processing. In addition, the antiviral Favipiravir inhibits RNA polymerase and is involved in entry blocking. Both drugs showed potential in vitro activity against SARS-CoV-2 [15].

Although at least six SARS-CoV-2 vaccines have been developed and licensed for emergency use, the safety, efficacy, durability and availability to large populations have not been established, so it is too early to know if COVID-19 vaccines will provide long-term protection. In addition, there is still no effective drug therapy for SARS-CoV-2. The current therapeutic strategies depend on supportive therapy and symptomatic management. Natural products can serve as prophylactic agents, halt virus progression, inhibit inflammatory cytokines secretion, and reduce infection, complications and mortality of SARS-CoV-2 [16].

Natural products have been a valuable source of therapeutic agents, molecules with therapeutic potentials, and an important source of more efficient drugs that are based on the chemical structure of natural products. For example, flavonoids have shown significant antiviral activities [17]. Curcumin and luteolin also show therapeutic potential against HIV targeting viral protease and HIV-1 transactivator of transcription [18,19]. Kaempferol also exhibits anti HSV-1 and 2 activities [20,21] *Aloe* is an ancient common plant species used as a medicinal plant. The genus *Aloe* comprises about 581 species. Its pharmacological properties and phytochemical characteristics have been extensively studied and evaluated [22]. Previous studies have shown that natural products from *Aloe* possess anti-inflammatory, immunostimulant, anti-cancer, antioxidant, anti-ageing, wound healing, antifungal, antibacterial and antiviral activities [23]. Natural products from *Aloe* showed antiviral and inhibitory activities against HSV-1 and 2, human cytomegalovirus (HCMV), influenza A, polio and other hemagglutinating viruses [23,24,25]. Aloin, a major compound of *Aloe* species, significantly reduces influenza viruses replication including oseltamivir-resistant (H1N1) influenza virus [26]. This indicates that *Aloe* genus offer a rich source of potential anti-viral compounds.

Virtual screening and molecular modeling studies showed potential therapeutic activities of some natural products in inhibiting SARS-CoV-2 proteins including the main protease (M^pro^), spike glycoprotein (S) and angiotensin converting enzyme-2 (ACE2) receptor which are promising potential therapeutic targets [22,27]. In the present study, we conducted computational screening and molecular dynamics study on a library of isolated molecules from *Aloe* genus, investigated the binding affinity of these compounds with SARS-CoV-2 main protease (M^pro^), spike glycoprotein (S) through molecular docking analysis. We found six potential inhibitors from *Aloe* genus that effectively bind to SARS-CoV-2 main protease (M^pro^), and three inhibitors that effectively bind spike glycoprotein receptor binding domain-ACE2 interface.

## 2. Results and Discussion

### 2.1. Phytochemical Constituents of Aloe

The tested library of active constituents from *Aloe* genus comprised phytochemicals that cover major classes of natural products (Figure 1). Phytochemical studies of the genus *Aloe* plants showed the presence of anthraquinones, chromones, coumarins, flavonoids, simple phenolic compounds, phenyl pyrans and phenyl pyrones, benzofurans, naphthalene derivatives, alkaloids and fatty acid derivatives (Table A1 and Figures S1–S18). Out of 237 compounds compiled in the library; anthraquinones were the most abundant constituents, with a percentage of 36.29%, followed by chromones (27.43%) and simple phenolic compounds (7.17%), while alkaloids, coumarin and fatty acid derivatives constituents were less abundant.

### 2.2. Structure-Based Virtual Screening and Molecular Docking of Aloe Phytochemicals on SARS-CoV-2 Spike Glycoprotein and Main Protease 

**Figure 2:**
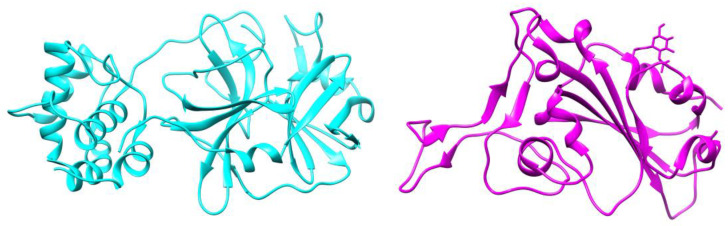
Three-dimensional crystal structure of the therapeutic targets of SARS-CoV-2 M^pro^ main protease (PDB ID: 6LU7, cyan) and spike glycoprotein (PDB ID: 6M0J, magenta).

**Figure 3:**
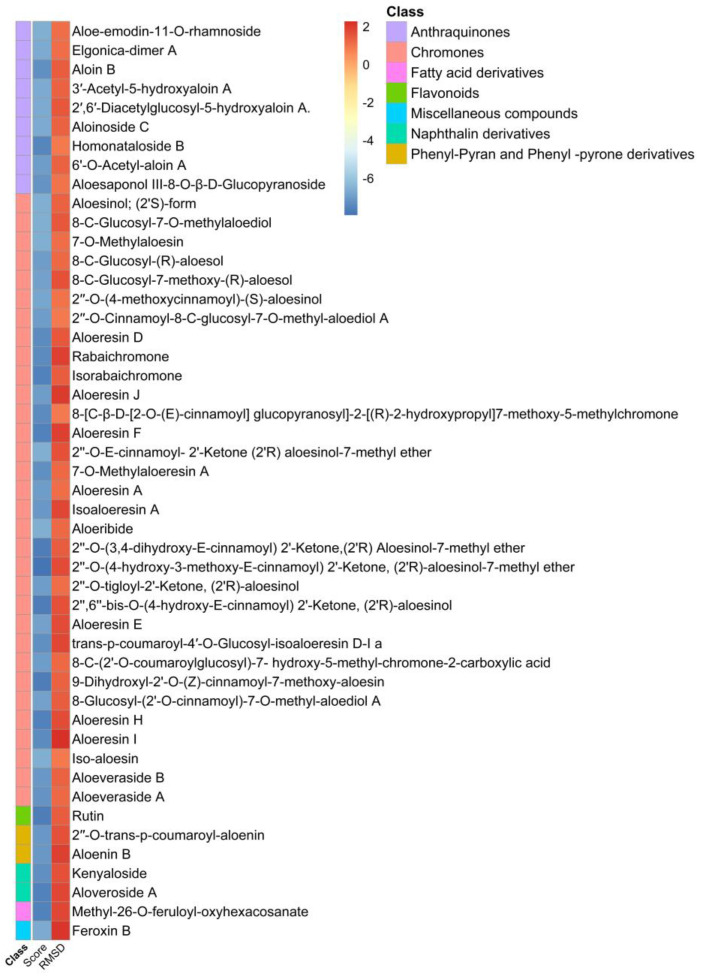
Docking scores and RMSD values of isolated compounds from *Aloe* genus against SARS-CoV-2 main protease M^pro^.

**Figure 4:**
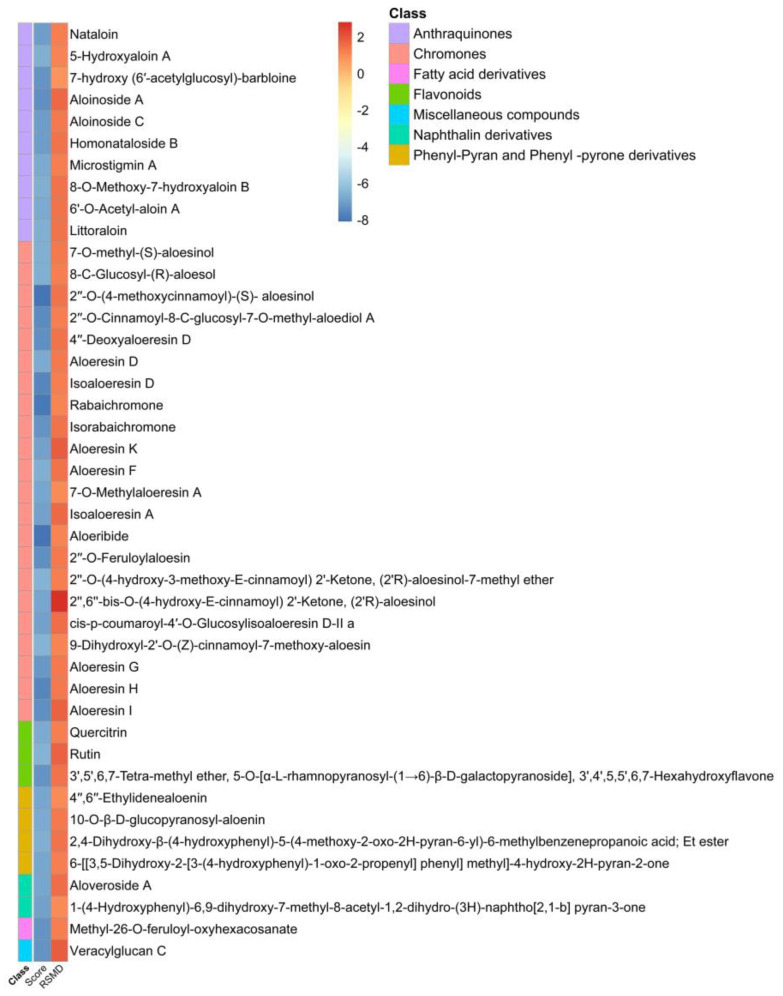
Docking scores and RMSD values of isolated compounds from *Aloe* genus against SARS-CoV-2 spike glycoprotein.

High-throughput virtual screening of compounds from *Aloe*, was followed by molecular docking and MD simulation. Since ligand binding to a protein of interest is the first step in drug discovery, molecular docking is widely used to predict and identify ligands that fit into the binding pocket of a protein of interest [28]. Our screening was performed against two major drug discovery and therapeutic targets of SARS-CoV-2, spike glycoprotein and M^pro^ proteins [7,12]. SARS-CoV-2 main protease M^pro^ is critical for the life cycle of the virus. Approximately, two thirds of the SARS-CoV-2 genome is translated into polyproteins pp1a and pp1ab, that are cleaved with M^pro^ into nonstructural proteins that are involved in the production of viral membrane, spike and nucleocapsid proteins [29]. M^pro^ is a dimer that has cysteine and histidine in the active site which form a catalytic dyad, conserved among coronaviruses making it an ideal therapeutic target [12]. In molecular docking studies, the ligand-receptor interaction with protein active site residues is established by formation of some interactions including hydrogen bonds, Van der Waal force interaction, π-sigma bond, π–π interaction, electrostatic interaction, and many other hydrophobic interactions. Hydrogen bonds are essential for interaction, lowering the binding energy and stabilizing the ligand-receptor docked complex. Pharmacologically, it is well-known that blockade of a receptor active site by a ligand terminates its functional activity [30]. Our molecular docking approach was validated by docking of hydroxychloroquine, a potent inhibitor of SARS-CoV-2 M^pro^. Hydroxychloroquine acts as a lysomotropic agent that inhibits viral entry and viral endocytosis. Viral entry and replication are highly dependent on the acidic pH of lysosomes and endosomes, and some host proteases which are also active in acidic pH (pH 5–5.5) [31]. Chloroquine and its analogues are diprotic weak bases that in their unprotonated forms, readily diffuse through cellular and organelle membranes such as lysosomes, endosomes and Golgi vesicles increasing pH from 6.3 to 6.7 [32,33,34]. In addition to disruption of endocytic pathway pH, chloroquine and hydroxychloroquine have been recently found to be potent inhibitors of SARS-CoV-2 M^pro^ but not viruses that belong to *Rhabdoviridae* [35]. In our study, the compounds previously isolated from *Aloe* plants were virtually screened against SARS-CoV-2 main protease M^pro^ (PDB ID: 6LU7) (Figure 2) and spike glycoprotein (PDB ID: 6M0J) (Figure 2) to find potential inhibitors for SARS-CoV-2. Using our docking approach, hydroxychloroquine interacted with SARS-CoV-2 protein M^pro^ and docked hydroxychloroquine bound to the active site with and RMSD of 1.2 Å. Molecular docking data were filtered to remove compounds with scores > −6.5 for both SARS-CoV-2 main protease M^pro^ (Figure 3 and Table A1) and spike glycoprotein (Figure 4 and Table A1). Molecular docking was performed by examining the interactions of these compounds with the active site residues of these proteins and analysis of results. 

**Figure 5:**
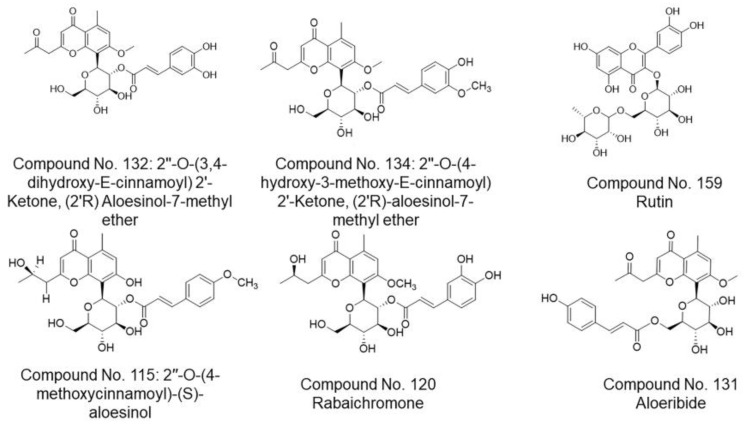
Chemical structures of top scoring active compounds from *Aloe* genus. Top panel, top scoring active compounds for SARS-CoV-2 protein M^pro^. Lower panel, top scoring active compounds for SARS-CoV-2 protein spike glycoprotein.

**Figure 6:**
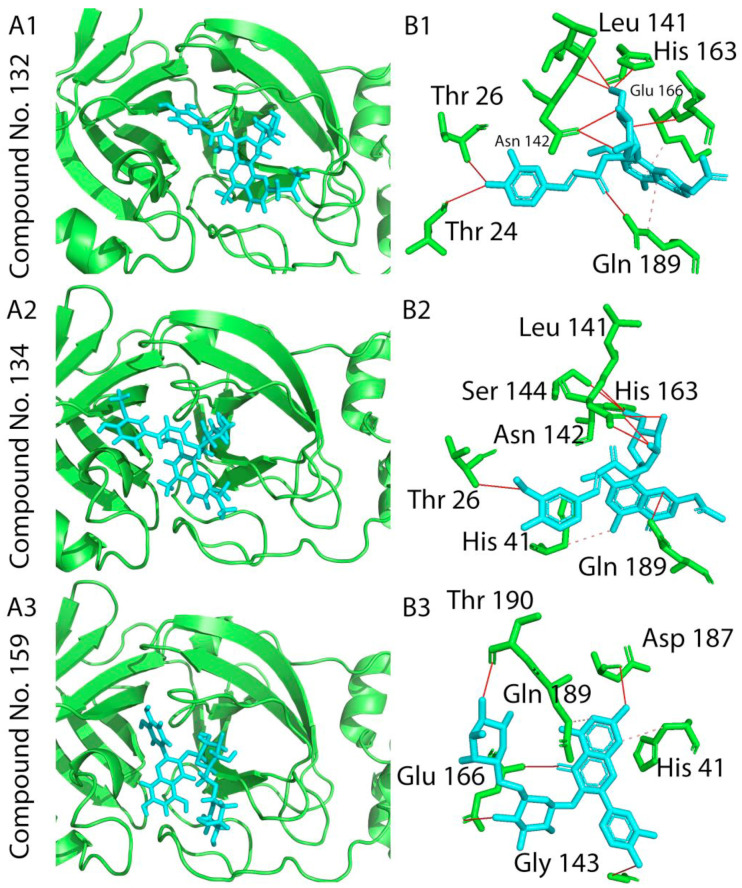
2D and 3D docking interactions complexes of compounds; **132** (**A1**,**B1**), 134 (**A2**,**B2**) and **159** (**A3**,**B3**) with SARS-CoV-2 main protease protein M^pro^. Solid red line: Hydrogen bonds. Dashed pink line: Hydrophobic interactions.

Compounds scoring lower than −5.00 kcal/mol are expected to be active. These compounds were then filtered by RMSD value [30], to evaluate experimental stability of the docked ligand conformers. RMSD values around 1.5 Å, are considered successful and stable while those beyond 2 Å indicate instability of ligand conformation and docking parameters [36]. For SARS-CoV-2 protein M^pro^, the binding energy observed for these compounds ranged from−7.950 to −0.339 kcal/mol while for spike glycoprotein, binding energy ranged from −8.088 to −5.437 kcal/mol. The top three scoring compounds for SARS-CoV-2 protein M^pro^ were compound **132** (2′-oxo-2′-O-(3,4-dihydroxy-*E*-cinnamoyl)-(2′*R*) aloesinol-7-methyl ether), compound **134** (2′-oxo-2′-O-(4-hydroxy-3-methoxy-(*E*)-cinnamoyl)-(2′*R*)-aloesinol-7-methyl ether) and compound **159** (rutin), (Table 1 docking scores and Figure 5, top panel). These three compounds showed the strongest interaction with the active site of SARS-CoV-2 protein M^pro^. Molecular 2D and 3D interactions complexes of compounds **132**, **134** and **159** with SARS- SARS-CoV-2 protein M^pro^ are shown in Figure 6.

On the other hand, the top three scoring compounds for SARS-CoV-2 spike glycoprotein were compounds compound **115** (2″-O-(4-methoxycinnamoyl)-(*S*)-aloesinol), compound **120** (rabaichromone), and compound **131** (aloeribide), (Table 1 docking score, and Figure 5, lower panel).

**Figure 7:**
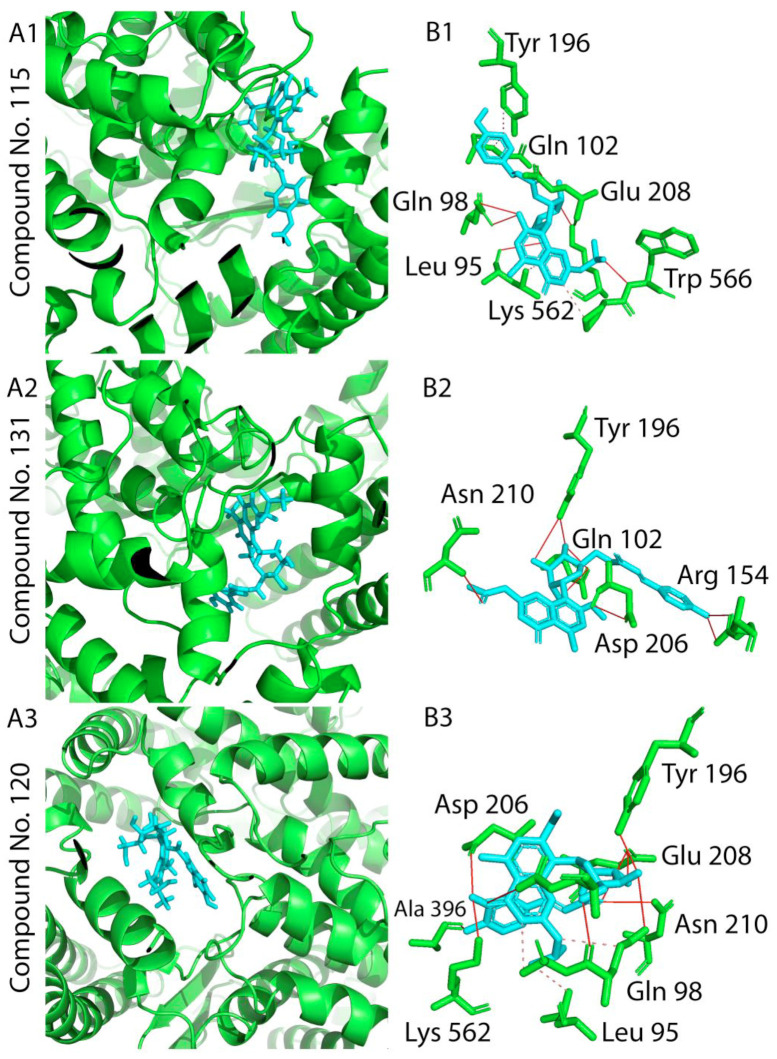
2D and 3D docking interactions complexes of compounds **115** (**A1**,**B1**), **131** (**A2**,**B2**), and **120** (**A3**,**B3**) with SARS-CoV-2 spike glycoprotein. Solid red line: Hydrogen bonds. Dashed pink line: Hydrophobic interactions.

These three compounds showed the strongest interaction with SARS-CoV-2 RBD. Molecular 2D and 3D interactions complexes of compounds **115, 120** and **131** with SARS- SARS-CoV-2 protein spike glycoprotein receptor binding domain are shown in Figure 7. In depth analysis showed that chromone derivatives **132** and **134** had high binding affinity as lead compounds for developing SARS-CoV-2 M^pro^ inhibitors. These compounds had a score of −7.683 kcal/mol (RSMD = 1.37) and −7.951 kcal/mol (RSMD = 1.72), respectively (Table 1). 

The interacting residues of SARS-CoV-2 M^pro^ involved in interactions with compound **132** were ASN142A, ASN142A, HIS163A, GLN189A, GLU166A and GLN189A (Figure 6A1,B1 and Table 1), while compound **134** interacted with ASN142A, ASN142A and HIS163 (Figure 6A2,B2 and Table 1). In addition, the flavonoid compound **159** forms a hydrogen bond with the M^pro^ protein THR190A amino acid residue with −7.728 kcal/mol as scoring value (Figure 6A3,B3 and Table 1). Regarding the interaction of the compounds with SARS-CoV-2 spike glycoprotein, this was mainly supported by hydrogen bonds, π-H, ionic and hydrophobic interactions. The highest scoring compounds were the chromone derivatives **115** (2″-*O*-(4-methoxycinnamoyl)-(*S*)-aloesinol), **120** (rabaichromone) and **131** (aloeribide) with binding energies −8.057, −7.871 and −8.088 kcal/mol, respectively. Compound **115** interacted with SARS-CoV-2 RBD TRP566A, LYS562A, LYS562A and VAL209 (Figure 7A1,B1 and Table 1). Moreover, compound **131** interacted with GLN102A, ASN210A and ASP206A (Figure 7A2,B2 and Table 1) by π i- H bond while compound **120** formed hydrogen bonds with ALA396A, ASP206A and GLU208A amino acid residues (Figure 7A3,B3 and Table 1).

### 2.3. Molecular Dynamics Simulation 

**Figure 8:**
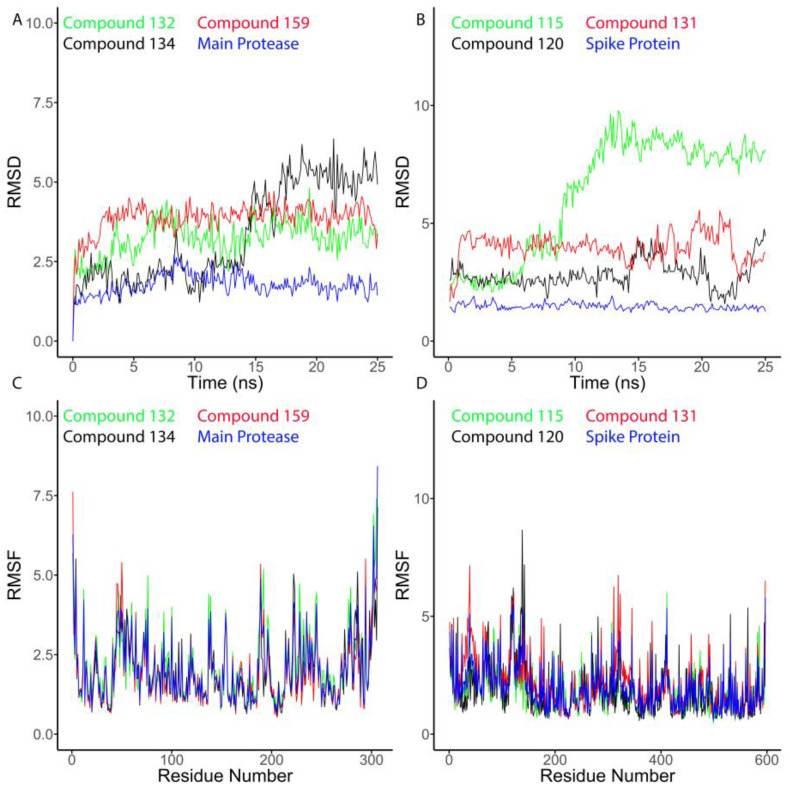
Analysis of the molecular dynamics (MD) simulations for top scoring compounds from *Aloe* genus. (**A**) RMSD analysis for M^pro^ and the ligands **132, 134** and **159**. (**B**) RMSD analysis for spike glycoprotein and the ligands **115**, **120** and **131**. (**C**) RMSF analysis for M^pro^ and the ligands **132**, **134** and **159**. (**D**) RMSF analysis for spike glycoprotein and the ligands **115**, **120** and **131**.

Conventional docking approaches do not account for the inherent protein binding site flexibility and the many protein conformational rearrangements [37]. Computational tools for drug discovery such as molecular dynamics take into account structural flexibility and entropic effects which produce accurate predictions of small molecule-protein binding thermodynamics and kinetics [38]. Hence dynamical docking considers flexibility of drug-protein binding and conformational changes, solvation of drug-protein complex and temperature [38,39]. Unbiased millisecond-long can predict spontaneous drug-protein entire binding [40]. In addition, recent developments in dynamical docking such as enhanced sampling for dynamical docking, path-based and alchemical transformations have greatly impacted drug discovery [38]. To validate molecular docking results, we subjected the top scoring compounds to unbiased molecular dynamics simulation experiments. The three top scoring M^pro^ inhibitor hits **132**, **134**, and **159** were able to achieve stable binding inside the active site with low deviations across the course of simulations (Average RMSD = 3.22, 3.32, and 3.86 Å, respectively) and convergent binding free energies (ΔG = −6.9, −6.8, and −6.5 kcal/mol, respectively), (Figure 8A). 

With respect to SARS-CoV-2 spike glycoprotein, both compounds **120** and **131** were stable inside the binding site during MD simulation, with scoring average RMSDs of 2.81 Å and 3.96 Å, respectively, and ΔG of −7.4 and −6.8 kcal/mol, respectively (Figure 8B). On the other hand, compound **115** was significantly less stable (average RMSD = 6.2 Å) inside the SARS-CoV-2 spike glycoprotein binding site, and this instability was further translated into a low binding free energy (ΔG = −4.5 kcal/mol) compared to compounds **120** and **131** (Figure 8B). RMSF is an expression of the average residual mobility throughout simulation in a structure and a higher RMSF value indicates more flexibility during MD simulation. We calculated the RMSF value for the top scoring compounds from *Aloe* genus with SARS-CoV-2 M^pro^ and SARS-CoV-2 spike glycoprotein and plotted RMSF value versus residue number (Figure 8C,D). The results indicate that compounds **159** and **120** had high RMSF values compared to other compounds. The RMSD and RMSF values indicate that the top scoring compounds from *Aloe* genus were stable and had greater random motion during the simulation. The inhibitors identified in in our docking analysis that showed interaction with SARS CoV-2 spike protein and M^Pro^ are in agreement with previously reported results [41]. Arokiyaraj et al. found that several polyphenolic compounds from *Geranii Herba*, including geraniin, kaempferitrin, quercitin, gallic acid, and kaempferol interacted with amino acid residues in the SARS-CoV-2 RBD active site inhibiting the interaction of SARS-CoV-2 RBD with ACE2. Arokiyaraj et al. also reported that these polyphenolic compounds interacted strongly with amino acids in the active site of SARS-CoV-2 M^pro^ and its proximity leading to blockade of the nucleophilic attack toward His 41 and blockade of proteolytic activity. In agreement with this, we found that quercetin interacted with SARS CoV-2 RBD and M^pro^ with binding energies of −5 Kcal/mol and −5.5 Kcal/mol respectively, similar to the results reported by Arokiyaraj et al. for quercetin interaction with RBD and M^pro^ −5.71 Kcal/mol and −6.49 kcal/mol, respectively. In addition, we found that gallic acid interacted with SARS CoV-2 RBD and M^pro^ with binding energies of −4.19 Kcal/mol and −3.56 Kcal/mol, respectively, similar to the binding energies reported by Arokiyaraj et al. for the gallic acid interaction with SARS CoV-2 RBD and M^pro^, −4.21 kcal/mol and −4.46 kcal/mol, respectively. These finding indicate that phenolic compounds from *Aloe* are potential inhibitors for SARS CoV-2 RBD and M^pro^ [41].

### 2.4. Drug like Properties, and Pharmacokinetic Prediction of the Ligands

Drug-like properties and pharmacokinetic properties are intrinsic characteristics of drugs that may need to be optimized independently from pharmacodynamics properties during drug development. It is a balance among molecular properties affecting pharmacodynamics and pharmacokinetics of small molecules. These molecular properties such as membrane permeability and bioavailability are always connected to some basic molecular descriptors such as lipophilicity log P, (Tendency of a compound to partition into an aqueous matrix versus lipid matrix), molecular weight (MW), topological polar surface area (TPSA), or hydrogen bond acceptors and donors count in a molecule. Lipophilicity impacts drug’s absorption, distribution, metabolism, elimination (ADME) and plasma protein binding properties. In addition, the number of hydrogen bond donors and hydrogen bond acceptors influence drug’s pKa (−log Ka). The solubility of small molecules impacts their bioavailability and the need for frequent dosing, hence we investigated the ADME properties, inhibition of cytochrome P450 (CYP), modulation of P-glycoprotein (Pgp), solubility, plasma protein binding and permeability of the top scoring compounds in our analysis. The best scoring compounds for both SARS-CoV-2 M^pro^ and spike glycoprotein were tested for obeying Lipinski’s rule of five parameters, which states that drugs having log P ranging from 0 to 5, have high possibility of oral absorption [42]. Data (Table 2) showed that the compounds have log P values that ranged from −1.06 to 2.8 that does not exceed 5.0 indicating reasonable probability of their good absorption. The number of hydrogen bond donors was variable and ranged from 4 to10 that is more than 5 and also hydrogen bond acceptors were 11–16 that is more than 10. All compounds have number of atoms that ranged from 40 to 43 which is within 20–70. In addition, the topological polar surface area (TPSA) of the compounds as parameter for the prediction of drug transport properties showed TPSA value greater than 140 Å^2^ tend to be poor at permeating cell membranes. Despite violation of some rules, approved anticancer and anti-infective drugs from natural products or their semisynthetic derivatives such as taxol and amphotericin B have also some violations but are biologically effective as drugs. Therefore, these results don’t interfere with the development of these compounds as potential SARS-CoV-2 therapeutic agents [43].

Data resulting from Prediction of ADME server (Table S1) revealed that compounds **115**, **120**, **131**, **132**, **134** may be better absorbed from the intestinal tract upon oral administration as they showed good human intestinal absorption (HIA) (77.110857, 57.614849, 82.803611, 66.184384 and 79.978975, respectively). Caco-2 cell permeability is a model for selecting drug candidates for oral administration [44]. All compounds showed medium Caco-2 predicted permeability and medium MDCK predicted cell permeability [45]. Moreover, all compounds showed moderate predicted plasma protein binding (PPB) (Table S1) except for compound **159**, which showed weak predicted PPB, which indicates predicted decreased excretion and increased predicted half-life. It is important to consider drug’s interaction with plasma proteins, transporters and CYP450s for the successful selection of drug candidate. CYP2C19 and CYP2C9 inhibition leads to increased drug plasma concentration, leading to potential side effects [46,47]. All top scoring compounds were predicted to inhibit CYP2C19 and CYP2C9. CYP2D6 metabolizes many drugs and toxins [48]. None of the top scoring compounds showed predicted inhibitory activity to CYP2D6. CYP3A4 is also involved in metabolism of xenobiotics and is highly expressed in the liver and intestine [49]. The six top scoring compounds were predicted to inhibit CYP3A4. Drug resistance is a major concern in drug development. Multidrug resistance is regulated by a network of ATP-binding cassette (ABC) proteins that detoxify xenobiotics and act as drug transporters and efflux pumps. P glycoprotein (Pgp; ABCB1) is the most popular and well-studied efflux pump [50,51]. Pgp has intrinsic ATPase activity to drive active transport and generate a concentration gradient leading to transport of drugs to the extracellular space and inhibition of drug activity [51]. Pgp is highly expressed in blood-brain barrier cell, liver, intestine and kidney. Thus, it is important to predict drug’s binding to Pgp. Only compound **115** was predicted to inhibit Pgp and hence it may affect the activity or excretion of other Pgp substrates. Compound **159** had the highest water solubility (217.207 mg/L) while the other five compounds had low water solubility, hence this should be considered during drug development. In addition, skin permeability is an important factor to consider during drug development for the potential of dermal drug delivery and risk assessment of drugs that may contact skin [52]. Skin permeability also increases drug’s plasma concentration and activity. It has been reported that logP between −3 to +6 predict drug’s skin permeability [53]. SKlogD, SKlogP and SKlogS are related to drugs’ skin permeability and lipophilicity. All the six top scoring compounds had skin permeability values ranging from −4.6 to −3.6, indicating that they may not be absorbed through skin and thus should not pose a dermal exposure risk. Finally, all compounds did not have predicted ability to pass the blood brain barrier (BBB) and are not expected to be neurotoxic.

## 3. Materials and Methods

### 3.1. Phytochemical Review of Genus Aloe

Intensive review of the literatures in ScienceDirect, PubMed, SciFinder and has been conducted to identify compounds from *Aloe* genus. 

### 3.2. Molecular Docking, Data Software and Visualization

#### 3.2.1. Preparation of Protein and Active Site Prediction

In this study, two SARS-CoV-2 proteins which facilitate viral–host interaction and replication were selected from the RCSB protein databank (https://www.rscb.org/pdb, accessed on 20 February 2021). The proteins are SARS-CoV-2 main protease (PDB ID: 6LU7, resolution = 2.16 Å) [54] and spike glycoprotein (PDB ID: 6M0J, resolution = 2.45 Å) [42]. The 3D protein structures were prepared using Molecular Operating Environment software (MOE 2014.0901) Ligx option. The site finder function used to calculate and predict possible active potential site of selected proteins for ligand binding in the receptor. PyMol 2.3 software was used for visualization.

#### 3.2.2. Preparation of Ligand

Reviewing the available literatures identified 237 phytochemical compounds that were isolated from genus *Aloe* (Table A1 and Figures S1–S18). All these molecular structures were imported to MOE and subjected to 3D protonation and energy minimization using MMFF94s force field and ligand database was constructed. Ligand coordinate files were extracted from PDB files and used as reference structures for root mean square deviation (RMSD) calculations.

#### 3.2.3. Docking Analysis

Flexible ligand-rigid receptor docking was performed with MOE-DOCK for molecular docking. The parameters of scoring were Triangle Matcher, scoring was set at London dG with 30 output poses and rescoring at GBVI/WSA dG retaining 10 refined poses. The docking score, root mean square deviation (RSMD), 2D and 3D interactions were recorded [55]. The results of docked ligands are chosen based on the most negative docking score. The docking score represents the best-bound ligand conformations and relative binding affinities. The best-docked conformations for comparison of the binding of the drugs and targets of SARS-CoV2 were selected based on number of hydrogen bonds, binding energy (kcal/mol), upper and lower bound RMSD, number of interacting residues, and forces which stabilized the receptor-ligand complex. RMSD and RMSF of the ligand interaction with the target protein were calculated using the following formulas:

(1) $$RMSD_{x}=\;\sqrt{\frac{1}{N}\sum_{i=1}^{n}\left(r_{i}^{t}\left(t_{x}\right)\right)-\;r_{i}(t_{ref\;}}){)}^{2}$$

(2) $$RMSF_{i}=\;\sqrt{\frac{1}{T}\sum_{t=1}^{T}<\left(r_{i}^{t}\left(t\right)\right)-\;r_{i}(t_{ref\;}}){)}^{2}>$$

where *N* is the number of atoms, *t_ref_* is the reference time, *r*′ is the position of the selected atoms in frame *x* after superimposing on the reference frame, frame x recorded at time *t_x_*, *T* is the trajectory time over which the RMSF was calculated, *r* is the position of an atom. Poses of docked compounds are automatically calculated by docking function in Molecular Operating Environment software.

### 3.3. Molecular Dynamics Simulation 

MD simulation experiments were performed as previously described [43]. Briefly, the Molecular Dynamics (NAMD) 2.6 software [45], employing the CHARMM27 force field [56] was used for simulations. Hydrogen atoms were added to initial coordinates of proteins using the psfgen plugin included in the Visual Molecular Dynamics (VMD) 1.9 software [57]. Subsequently, the protein systems were solvated using TIP3P water particles and 0.15 M NaCl. The equilibration procedure comprised 1500 minimization steps followed by 30 ps of MD simulation at 10 K with fixed protein atoms. Then, the entire systems were minimized over 1500 steps at 0 K, followed by gradual heating from 10 to 310 K using temperature reassignment during the initial 60 ps of the 100 ps equilibration MD simulation. The final step involved NTP simulation (30 ps) using the Nose–Hoover Langevin piston pressure control at 310 K and 1.013 bars for density (volume) fitting [58]. Thereafter, the MD simulation experiments were continued for 25 ns for the entire systems (20 ns for the enzyme–ligand complexes). The trajectory was stored every 0.1 ns and further analyzed with the VMD 1.9 software. The MD simulation output over 25 ns provided several structural conformers that were sampled every 0.1 ns (250 poses) to evaluate conformational changes of the entire protein structure to analyze the RMSD. All parameters and topologies of the compounds selected for MD simulation were prepared using the online software Ligand Reader & Modeler [59] and the VMD Force Field Toolkit (ffTK) [57]. Binding free energy calculations (∆G) were performed using the free energy perturbation (FEP) method through the web-based software Absolute Ligand Binder [60] together with MD simulation software NAMD 2.6 [45]. Hydrogen bonds and hydrophobic interactions between protein and ligand were also analyzed using Protein-Ligand Interaction Profiler [61].

### 3.4. Drug Like Properties, and ADME Prediction of the Ligands

The drug likeliness of best pose scoring compounds is specified by the Lipinski’s rule and molecular properties prediction was calculated by the free access website https://www.molinspiration.com/cgi-bin/properties, accessed on 20 February 2021. ADME Prediction were determined by PreADMET estimation server website [62].

## 4. Conclusions

In recent years, advances in computational resources and software tools led to emergence of molecular dynamics (docking and scoring tool), as the first phase in drug screening and discovery. In addition, absence of new cell culture models for working safely with highly pathogenic viruses makes virtual screening, docking and dynamics of great importance. *Aloe* genus is a rich source of phytochemicals with a wide range of therapeutic activity. Several natural products from *Aloe* have shown strong antiviral activity, inhibiting replication and entry and of HSV-1 and 2, human cytomegalovirus (HCMV), influenza A and polio. Aloin significantly reduces replication of oseltamivir-resistant (H1N1) influenza virus. In our study, we applied computational screening of 237 natural product compounds from *Aloe* genus and identification of six compounds as stable potential inhibitors of SARS-CoV-2 main protease and spike glycoprotein. Our molecular docking analysis showed that theses six compounds are stable and safe. Compounds **132**, **134** and **159** were the top three scoring potential inhibitors of for SARS-CoV-2 main protease. These compounds interacted strongly with amino acids in the active site of SARS-CoV-2 main protease. Rutin (**154**) is known to have antiviral activity against influenza virus [63]. Compounds **115**, **120** and compound **131** were the top scoring potential inhibitors of SARS-CoV-2 spike glycoprotein. The results highlighted chromone derivatives as potential inhibitors for SARS-CoV-2 according to their best scores of binding affinities to the mentioned target proteins among the examined compounds. The results of this in-silico investigation (docking and molecular dynamics simulation) should have a great impact for drug repurposing studies. In the future, in vitro, in vivo and clinical studies shall be conducted to further validate the effectiveness of these compounds as potential treatments for COVID-19 and to identify compounds with best pharmacokinetic profiles. In addition, it will be of great importance to apply newly-developed algorithms and utilize the development of steered molecular dynamics for evaluating the binding of the top scoring compounds to SARS-CoV-2 target proteins [64].

## Figures and Tables

**Figure 1:**
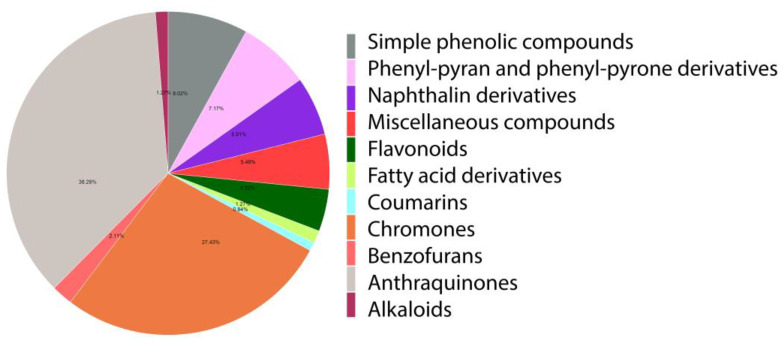
The percentage of different classes of phytochemicals reported from the genus *Aloe*. Anthraquinones 36.3%, chromones 27.4%, coumarin 0.8%, flavonoids 4.2%, simple phenolic compounds 8%, phenyl pyran and phenyl pyrone derivatives 7%, benzofurans 2%, naphthalene derivatives 5.9%, alkaloids 1.2%, fatty acid derivatives 1.2% and miscellaneous compounds 5.5%.

**Table 1:** Molecular docking results and interactions of the three top scoring compounds of *Aloe* species with SARS-CoV-2 proteins.

Protein	No.	Docking Score (kcal/mol)	RSMD ^1^ Refine	CLogP	Receptor	Interaction	Distance	E (kcal/mol)
main Protease (PDB ID: 6LU7)	**132**	−7.68	1.37	0.25	ASN142A	H-donor	3.01	−1.9
					ASN142A	H-donor	2.81	−2.5
					HIS163A	H-acceptor	3.35	−0.7
					GLN189A	H-acceptor	3.03	−1.4
					GLU166A	pi-H	4.42	−0.7
					GLN189A	pi-H	3.62	−0.6
	**134**	−7.95	1.72	0.69	ASN142A	H-donor	3.44	−0.6
					ASN142A	H-donor	2.78	−2
					HIS163A	H-acceptor	3.29	−1.2
	**159**	−7.72	1.40	−1.36	THR190A	H-donor	2.94	−0.8
Spike Glycoprotein (PDB ID: 6M0J)	**115**	−8.05	1.49	1.21	TRP566A	H-acceptor	2.93	−2.4
					LYS562A	H-acceptor	3.11	−12.2
					LYS562A	Ionic	3.11	−3.8
					VAL209	pi-H	4.17	−0.6
					VAL209	pi-H	4.27	−0.6
	**131**	−8.08	1.13	1.02	GLN102A	H-donor	3.01	−1.2
					ASN210A	H-acceptor	3.31	−0.8
					ASP206A	pi-H	4.26	−0.7
	**120**	−7.87	1.17	0.46	ALA396A	H-donor	2.56	−0.5
					ASP206A	H-donor	2.78	−3.0
					GLU208A	H-donor	2.77	−1.1

^1^ RMSD; Root mean square deviation.

**Table 2:** Drug-likeness and molecular properties of top scoring compounds predicted at molinspiration server. miLogP: Molinspiration LogP (Octanol-water partition coefficient); TPSA: Total polar surface area (drug transport properties); natoms-Number of atoms; Mol.Wt (g/mol): Molecular weight; nON: number of hydrogen bond acceptors; nOHHN: number of hydrogen bond donors; nviolations: Number of Lipinski’s rule of five parameters violations; nrotb: Number of Rotatable Bonds (molecular flexibility).

	115	120	131	132	134	159
miLogP	2.8	1.84	2.15	1.66	1.96	−1.06
TPSA	176.12	196.35	172.97	193.19	182.2	269.43
natoms	40	41	40	41	42	43
MW	556.56	572.56	554.55	570.55	584.57	610.52
nON	11	12	11	12	12	16
nOHNH	5	6	4	5	4	10
nviolations	2	3	2	2	2	3
nrotb	9	9	9	9	10	6
volume	486.85	494.87	480.99	489.01	506.53	496.07
%ABS	48.23	41.25	49.32	42.34	46.14	16.04

## Data Availability

Not applicable.

## Supplementary Materials

The following are available online. Figures S1–S18: Compounds isolated from genus *Aloe*, Table S1: Predicted pharmacokinetics of top scoring compounds.

## Sample Availability

Samples of the compounds are not available from the authors.

## Appendix A

**Table A1:** Docking score of isolated compounds from Aloe plants against selected SARS CoV-2 proteins.

No.	Name of the Compound	Main Protease (PDB ID: 6LU7)		Spike Glycoprotein (PDB ID: 6M0J)		Plant Source	Ref.
		Score	RSMD	Score	RSMD		
I-Anthraquinones:							
**1**	Chrysophanol	−5.4584	0.8734	−5.4447	0.8709	*A. pulcherrima, A. dawei, A. megalacantha, A. vera*	[65,66,67,68]
**2**	8-*O*-Methylchrysophanol	−5.0678	0.4878	−4.8406	0.9352	*A. dawei*	[66]
**3**	Aloe-emodin	−5.3348	0.8782	−6.0546	0.9449	*A. megalacantha, A. arborescens A. vera, A. ferox*	[67,68,69,70,71,72]
**4**	7-Hydroxy-aloe-emodin	−5.3660	1.3842	−4.8938	0.9332	*A. succotrina*	[73]
**5**	Nataloe-emodin	−5.5017	0.7836	−5.2599	0.4924	*A. nyeriensis*	[74]
**6**	Mono-*O*-methyl-nataloe-emodin	−4.8059	1.2584	−4.6113	0.9158	*A. speciosa*	[74]
**7**	Emodin	−4.4060	0.9758	−4.8558	1.3623	*A. vera, A. spp*	[72,75,76,77]
**8**	Saponarin II	−4.7520	1.0190	−5.1119	1.3501	*A. megalacantha, A. pulcherrima, A. dawei, A. saponaria.*	[65,66,67,78,79,80]
**9**	Saponarin I	−5.5636	0.8416	−5.2625	1.3226	*A. megalacantha, A. pulcherrima, A. saponaria.*	[65,67,78,81]
**10**	Saponarin III	−5.5941	0.9280	−5.9907	0.9256	*A. megalacantha*	[67]
**11**	Helminthosporin	−5.2698	0.6476	−5.3963	1.3211	*A. megalacantha, A. dawei, A. Saponaria*	[66,67,79]
**12**	5-*O*-Methylziganein	−5.6054	0.8884	−3.3209	0.8923	*A. hijazensis*	[82]
**13**	Isoxanthorin	−4.7748	1.2234	−3.9051	0.9124	*A. Saponaria*	[79,80]
**14**	Deoxyerythrolaccin	−4.7968	1.3812	−5.2572	1.2640	*A. ferox, A. Saponaria*	[71]
**15**	Laccaic acid D Methyl ester	−6.1620	0.9956	−5.3786	0.8172	*A. Saponaria, A. dawei*	[66,78]
**16**	Madagascin	−4.2212	1.1677	−4.0269	0.9379	*A. vera*	[83]
**17**	3-Geranyloxyemodine	−5.4161	1.4882	−4.8861	1.1771	*A. vera*	[83]
**18**	Aloetinic acid.	−5.6006	1.6111	−2.8968	1.3504	*A. vera*	[84]
**19**	Nataloe-emodin-2-*O*-*β*-D-Glucopyranoside	−5.3915	0.9107	−4.4116	1.4211	*A. nyeriensis*	[85]
**20**	Aloe-emodin-11-*O*-rhamnoside	−6.5513	1.1442	−6.3233	1.2130	*A. vera*	[72,86]
**21**	1,1′,8,8′-Tetrahydroxy-3,3′-dimethyl-4,7′-bianthracene-9,9′,10 (10′*H*)-trione	−6.2519	0.8628	−3.7287	1.2329	*A. Saponaria*	[81]
**22**	Asphodelin	−5.8474	0.9813	−6.1966	1.2760	*A. megalacantha, A. Saponaria*	[67,81]
**23**	(1,1′,8,8′,10-Pentahydroxy-3,3′-dimethyl-10,7′-bianthracene-9,9′,10′-trione)	−5.2521	1.3390	−6.4419	1.2682	*A. Saponaria*	[81]
**24**	10-(chrysophanol-7′-yl)-10-hydroxychrysophanol-9-anthrone	−6.0946	1.6603	−5.7400	1.2765	*A. megalacantha*	[67]
**25**	Chrysalodin	−6.3819	1.4365	−5.8787	1.3680	*A. megalacantha*	[67]
**26**	10-*O*-Methylchrysalodin	−5.1180	0.9839	−5.1616	1.0078	*A. megalacantha*	[67]
**27**	Elgonica-dimer A	−6.6933	1.1067	−2.5862	1.4349	*A. elgonica, A. vera*	[87,88,89]
**28**	Elgonica-dimer B	−6.4416	1.2142	5.4378	1.6013	*A. elgonica, A. vera*	[87,88,89]
**29**	1,4′,5′,8,9′-Pentahydroxy-2′,6-dimethyl[2,9′-bianthracene]-9,10′(9′*H*,10*H*)-dione	−6.1595	1.1344	−6.2983	1.4995	*A. Saponaria*	[81]
**30**	Aloin A	−6.1758	1.0225	−5.8047	1.3500	*A. calidophila, A. schelpei, A. vera, A. perryi, A. ghibensis, A. gilbertii, A. trigonantha*	[68,69,90,91,92,93,94,95,96]
**31**	Aloin B	−7.2794	1.3710	−6.3227	1.1594	*A. vera, A. perryi, A. ghibensis, A. gilbertii,* *A. trigonantha*	[68,69,90,91,92]
**32**	7-Hydroxy-8-*O*-methylaloin A	−6.4555	1.4506	−6.1034	1.1466	*A. vera*	[97]
**33**	7-Hydroxy-8-*O*-methylaloin B	−3.3579	1.4722	−5.1135	1.1263	*A. vera*	[97]
**34**	Nataloin	−3.1203	0.9706	−7.1048	1.2243	*A. nyeriensis*	[85]
**35**	6′-*O*-Malonylnataloin	−6.1541	1.3288	−3.4142	1.0014	*A. ellenbeckii*	[98]
**36**	Homonataloin A	−3.4695	1.0697	−5.4443	1.4948	*A. lateritia, A. distans, A. cremnophila, A. citrina, A. vera*	[92,99,100]
**37**	Homonataloin B	−4.9349	1.4914	−4.9807	0.8487	*A. lateritia, A. excelsa, A. vera, A. perryi*	[92,99,101,102]
**38**	5-Hydroxyaloin A	−4.8766	1.1457	−6.6159	1.1690	*A. nobilis, A microstigma*	[99,103,104]
**39**	7-Hydroxyaloin A	−3.0164	1.4360	−5.5526	0.9072	*A. ghibensis, A. succotrina*	[73,91]
**40**	3′-Acetyl-5-hydroxyaloin A	−6.6767	1.3118	−5.1832	1.2296	*A. nobilis*	[104]
**41**	6′-Acetylglucosyl-5-hydroxyaloin A	−5.3535	1.3456	−5.4990	1.1070	*A. marlothii,* *Aloe rupestris*	[105]
**42**	7-Hydroxy (6′-acetylGlucosyl)-aloin	−5.2629	1.2352	−6.4008	1.4160	*A. succotrina*	[73,106]
**43**	7-hydroxy (6′-acetylglucosyl)-barbloine	−4.2565	1.4076	−7.2279	0.8115	*A. succotrina*	[73]
**44**	2′,6′-Diacetylglucosyl-5-hydroxyaloin A.	−6.7157	1.5569	−5.8313	1.3241	*A. nobilis*	[104]
**45**	4′,6′-Diacetylglucosyl-5-hydroxyaloin A	−4.9825	1.7716	−5.7746	1.0670	*A. nobilis*	[104]
**46**	8-*O*-Methoxy-7-hydroxyaloin A	−5.2694	0.8441	−1.2609	1.3127	*A. vera, A. trigonantha*	[92,93]
**47**	4′,6′-O-diacetate-7-Hydroxyaloin A	−1.0169	1.1487	−3.6664	1.1714	*A. succotrina*	[73]
**48**	4′,6′-O-diacetate-7-Hydroxyaloin B	−5.9283	1.8131	−3.8189	1.1443	*A. succotrina*	[73]
**49**	Aloinoside A	−5.5327	1.6795	−7.3722	1.6129	*A. ferox, A. spp.*	[92,94,95,107]
**50**	Aloinoside B	−4.2386	2.0931	−6.1225	0.9157	*A. vera, A. perryi*	[69,72,86,92,107,108]
**51**	Aloinoside C	−6.7035	1.3302	−7.0975	1.2894	*A. spp.*	[69]
**52**	Homonataloside B	−7.4548	0.9307	−7.0966	1.3986	*A. spp.*	[109]
**53**	Microdontin A	−5.3854	1.8687	−3.0568	1.3618	*A. gilbertii, A. microdonta, A. calidophila, A. vera, A. perryi, A. schelpei*	[92,94,95,96,110]
**54**	Microdontin B	−6.4459	1.4075	−6.2021	1.4041	*A. vera, A. perryi, A. microdonta*	[92,110]
**55**	Microstigmin A	−6.2212	1.2076	−6.7073	1.1774	*A. microstigma, A. broomii*	[103]
**56**	Desoxyaloin	−5.1323	1.4371	−5.4503	1.1224	*A. spp.*	[69]
**57**	8-*O*-Methoxy-7-hydroxyaloin B	−4.9067	1.2985	−6.6635	1.4981	*A. vera, A. trigonantha*	[92,93]
**58**	7-Hydroxyaloin B	−5.7138	1.6502	−6.2245	0.5540	*A. ghibensis, A. vera, A. succotrina*	[73,91]
**59**	6′-*O*-Acetyl-aloin B	−6.2829	1.1810	−6.2769	1.2832	*A. vera,* *A. trigonantha*	[93,111]
**60**	6′-*O*-Acetyl-aloin A	−7.0170	1.3290	−6.7174	1.4200	*A. vera, A. trigonantha*	[93,111]
**61**	6′-*O*-Acetyl-10-hydroxyaloin B	−5.4305	1.3206	−5.1675	1.1849	*A. claviflora*	[112]
**62**	10-Hydroxyaloin A	−5.5235	1.2517	−5.3937	0.9974	*A. vera*	[68,72,86,97]
**63**	10-Hydroxyaloin B	−4.5207	1.1644	−6.2037	1.4416	*A. vera,* *A. littoralis*	[68,72,97,113]
**64**	Aloinoside D	−4.0281	1.5439	−5.7067	1.6872	*A. sp.*	[69]
**65**	Deacetyllittoraloin	−5.4766	0.8960	−3.2865	1.2900	*A. littoralis*	[114]
**66**	Littoraloin	−6.7095	1.9848	−6.6415	1.4619	*A. littoralis*	[114]
**67**	Littoraloin	−5.4568	1.3628	−1.6450	1.3238	*A. littoralis*	[113]
**68**	Deacetyllittoraloin	−4.6536	1.4897	−3.4045	1.2748	*A. littoralis*	[113]
**69**	Littoraloside	−5.1344	1.6895	−5.7699	1.5576	*A. littoralis*	[114]
**70**	6,8-Dihydroxy-4-methylbenzanthrone	−4.8093	0.9928	−3.6017	1.1112	*A. vera*	[115]
**71**	Anthrone; Enol-form	−3.7149	1.7269	−3.3277	1.7661	*A. vera*	[116]
**72**	3,4-Dihydro-3,5,7-trihydroxy-9-methyl-1(2*H*)-anthracenone	−5.3077	0.5534	−5.5367	1.5708	*A. vera*	[117]
**73**	Aloesaponol II	−5.5596	0.9169	−4.0884	1.1997	*A. saponaria*	[78,80]
**74**	Aloesaponol II-6-methyl ether	−5.4278	0.8931	−5.6829	0.8923	*A. dawei*	[66]
**75**	Aloesaponol IV	−5.3144	1.0041	−5.8792	1.2443	*A. saponaria*	[79]
**76**	Aloesaponol I	−6.2731	0.6260	−5.5268	1.1253	*A. megalacantha, A. dawei, A. saponaria*	[66,67,78,80]
**77**	Aloesaponol III	−5.2375	1.6252	−2.8273	0.5258	*A. saponaria*	[79,80]
**78**	8-*O*-Methyl-aloesaponol III	−4.7557	2.6159	−5.4051	0.7606	*A. saponaria*	[79]
**79**	Aloesaponol III-8-*O*-*β*-D-Glucopyranoside	−7.1900	0.9527	−4.9331	1.1900	*A. saponaria*	[79,80]
**80**	Aloesaponol IV-8-*O*-*β*-D-Glucopyranoside	−6.4329	0.9875	−6.1878	1.0722	*A. saponaria*	[79]
**81**	*O*-de-Methylaloesaponol IV-4-Epimer, 4-*O*-*β*-D-glucopyranoside	−5.2621	1.6257	−6.0435	1.6168	*A. vera*	[118,119]
**82**	Aloesaponol IV-4-Epimer, 4-*O*-*β*-D-glucopyranoside	−5.5878	1.4697	−4.4875	1.2372	*A. vera*	[118,119]
**83**	Aloesaponol II-6-glucoside	−5.5097	1.6405	−6.3430	1.7211	*A. saponaria*	[78,80]
**84**	Aloesaponol I-6-*O*-*β*-D-Glucoside	−6.4018	1.0686	−6.4720	0.8722	*A. saponaria*	[78,80]
**85**	Prechrysophanol	−5.0097	1.3074	−4.9503	0.9273	*A. graminicola*	[120]
**86**	Aloechrysone	−5.5183	1.0812	−5.2203	1.1941	*A. berhana*	[121]
II-Chromones:							
**87**	2,7-Dihydroxy-5-methylchromone	−4.1044	1.5749	−4.3380	2.1225	*A. arborescens*	[122]
**88**	Altechromone A	−4.3964	1.3753	−4.0177	2.5253	*A. vera, A. ferox*	[76]
**89**	7-Hydroxy-5-(hydroxymethyl)-2-methylchromone	−4.6654	1.0133	−4.0034	1.0116	*A. vera, A. spp.*	[68,69,123]
**90**	5-(Hydroxymethyl)-7-methoxy-2-methylchromone	−5.0942	1.7087	−4.3604	0.6077	*A. vera*	[68]
**91**	Aloesone	−5.3434	0.8671	−4.4459	1.6654	*A. spp.*	[124]
**92**	Aloesol	−5.4824	1.1217	−4.5119	1.3491	*A. spp.*	[124]
**93**	Saikochromone A	−4.0246	1.1592	−4.6675	0.8802	*A. vera*	[68]
**94**	2−Carboxyethenyl-5,7-dihydroxychromone	−5.4560	0.8633	−4.3033	1.4776	*A. cremnophila*	[125]
**95**	5-((4E)-2′-Oxopentenyl)-2-hydroxymethylchromone	−5.2408	1.2458	−4.6543	0.9525	*A. vera*	[68]
**96**	2-Acetonyl-7-hydroxy-8-(3-hydroxyacetonyl)-5-methylchromone	−6.1295	1.1673	−5.6294	0.8828	*A. ferox*	[126]
**97**	5-((*S*)-2′-Oxo-4′-hydroxypentyl)-2-hydroxymethylchromone	−5.6533	0.8672	−4.5742	1.0536	*A. spp., A. vera*	[68,69,123]
**98**	7-O-methyl-(*R*)-aloesinol	−5.7749	1.3751	−6.3349	1.0728	*A. capensis, A. rubroviolacea, A. spicata*	[69,123,127,128]
**99**	7-O-methyl-(*S*)-aloesinol	−4.9862	1.0634	−6.5826	1.3861	*A. vera*	[129,130,131]
**100**	Aloesinol; (2′*S*)-form	−6.5412	1.3314	−5.9231	0.9830	*A. vera*	[129,130,131]
**101**	8-*C*-Glucosyl-7-*O*-methylaloediol	−6.5616	1.4702	−6.1983	1.3370	*A. vera*	[130,131,132]
**102**	*C*-2′-Decoumaroyl-aloeresin G	−6.3203	2.2082	−5.6674	0.6253	*A. vera, A. spp.*	[69,123,132]
**103**	Deacetylaloesin	−5.4580	1.4439	−5.6769	0.7237	*A. vera var. chinensis*	[133]
**104**	Isobiflorin	−4.3917	1.0141	−5.5361	1.3146	*A. vera*	[131]
**105**	8-*C*-Glucosyl-noreugenin	−6.0155	0.9498	−5.3311	1.2871	*A. vera*	[132]
**106**	Neoaloesin A	−4.1314	1.4402	−5.3727	1.2553	*A. vera*	[132,134]
**107**	Aloesin (Aloeresin B)	−5.1920	1.4721	−6.0011	0.9641	*A. vera, A. monticola, A. trigonantha A. saponaria, A. arborescens*	[68,92,93,132,135,136,137]
**108**	7-*O*-Methylaloesin	−6.5211	1.0562	−4.7873	0.7405	*A. vera, A. rupestris*	[92,105]
**109**	2-Acetonyl-8-(2-furoylmethyl)-7-hydroxy-5-methylchromone	−5.5785	1.2539	−5.9486	1.4100	*A. ferox*	[126]
**110**	8-*C*-Glucosyl-(*R*)-aloesol	−6.9810	1.2199	−6.5885	1.2379	*A. vera*	[68,92,132,136]
**111**	8-*C*-Glucosyl-(*S*)-aloesol	−5.9979	1.2224	−3.0178	1.2511	*A. vera*	[132]
**112**	8-*C*-Glucosyl-7-methoxy-(*R*)-aloesol	−6.9122	1.5762	−5.2251	0.8301	*A. vera*	[132]
**113**	8-*C*-Glucosyl-7-methoxy-(*S*)-aloesol	−6.2867	1.2856	−4.9903	0.9918	*A. vera*	[68,132,136]
**114**	2″-*O*-*p*-Coumaroyl-(*S*)-aloesinol	−6.4949	1.1099	−6.3880	1.7012	*A. nobilis*	[138]
**115**	2″-*O*-(4-methoxycinnamoyl)-(*S*)-aloesinol	−6.8222	0.9924	−8.0574	1.4996	*A. nobilis*	[104]
**116**	2″-*O*-Cinnamoyl-8-*C*-glucosyl-7-*O*-methyl-aloediol A	−6.9842	0.9200	−7.4491	1.1903	*A. vera*	[131]
**117**	4″-Deoxyaloeresin D	−1.9659	1.4660	−7.3969	1.4416	*A. vera*	[139]
**118**	Aloeresin D	−7.3865	1.5512	−6.6808	1.3432	*A. ferox, A. vera*	[69,123,132,140,141]
**119**	Isoaloeresin D	−5.8280	1.4838	−7.5561	1.2248	*A. vera*	[68,92,129,130,131,132,136]
**120**	Rabaichromone	−7.3914	1.9603	−7.8715	1.1703	*A. rabaiensis, A. vera*	[132,142]
**121**	Isorabaichromone	−7.5470	1.4108	−7.2656	1.4428	*A. vera*	[129,130,131,132]
**122**	Aloeresin J	−6.9723	1.9908	−6.4352	1.4979	*A. vera*	[132]
**123**	8-[*C*-*β*-D-[2-*O*-(E)-cinnamoyl] glucopyranosyl]-2-[(R)-2-hydroxypropyl]7-methoxy-5-methylchromone	−7.3516	0.9022	−5.8935	1.2315	*A. vera*	[132]
**124**	Aloeresin K	−6.2857	1.5121	−6.9020	1.8499	*A. vera*	[111,132]
**125**	Aloeresin F	−7.5814	1.9617	−6.6320	1.4268	*A. peglerae*	[143,144]
**126**	2″-*O*-*E*-cinnamoyl-2′-Ketone (2′*R*) aloesinol-7-methyl ether	−6.5227	1.6510	−5.7926	1.2582	*A. broomii*	[145]
**127**	7-*O*-Methylaloeresin A	−7.3061	1.1520	−6.8482	0.9873	*A. vera, A. perryi, A. marlothii*	[92,105,132]
**128**	Aloeresin A	−7.0228	1.1297	−6.1730	1.3563	*A. cremnophila, A. jacksonii, A. arborescens, A. vera*	[92,132,146]
**129**	Isoaloeresin A	−7.0698	1.8469	−6.9601	1.6099	*A. ferox*	[147]
**130**	6″-*O*-*p*-Coumaroylaloesin	−6.1054	1.3654	−5.5547	1.4791	*A. vera, A. castanea*	[143,144]
**131**	Aloeribide	−6.5988	1.1683	−8.0886	1.1338	*A. vera, A. monticola*	[86]
**132**	2″-*O*-(3,4-dihydroxy-*E*-cinnamoyl) 2′-Ketone, (2′*R*) *Aloe*sinol-7-methyl ether	−7.6830	1.3783	−5.1857	0.8784	*A. broomii*	[145]
**133**	2″-*O*-Feruloylaloesin	−6.3246	1.4172	−7.3571	1.3775	*A. arborescens*	[148]
**134**	2″-*O*-(4-hydroxy-3-methoxy-*E*-cinnamoyl) 2′-Ketone, (2′*R*)-aloesinol-7-methyl ether	−7.9510	1.7236	−6.5096	1.2159	*A. africana*	[145]
**135**	2″-*O*-tigloyl-2′-Ketone, (2′*R*)-aloesinol	−7.0060	1.0927	−1.4865	1.2366	*A. cremnophila, A. jacksonii*	[125]
**136**	2″,6″-bis-*O*-(4-hydroxy-*E*-cinnamoyl) 2′-Ketone, (2′*R*)-aloesinol	−7.6466	1.6059	−6.8589	2.8077	*A. speciosa*	[145]
**137**	Aloeresin E	−6.8483	1.7166	−3.3935	1.5214	*A. peglerae*	[143,144]
**138**	Aloeresin C	−3.1926	1.4036	4.6545	1.2671	*A. spp.*	[140,141]
**139**	*trans-p-*coumaroyl-4′-*O*-Glucosyl-isoaloeresin D-I a	−7.3070	1.8254	−5.2510	1.8902	*A. vera*	[129,130,131,132]
**140**	*cis-p*-coumaroyl-4′-*O*-Glucosylisoaloeresin D-II a	−5.1214	1.9414	−6.9804	1.5954	*A. vera*	[129,130,131,132]
**141**	8-*C*-(2′-*O*-coumaroylglucosyl)-7-hydroxy-5-methyl-chromone-2-carboxylic acid	−7.0093	1.2428	−3.8621	1.2324	*A. vera, A. perryi*	[92]
**142**	9-Dihydroxyl-2′-*O*-(*Z*)-cinnamoyl-7-methoxy-aloesin	−7.6616	1.3432	−6.5510	1.0865	*A. vera*	[132]
**143**	5-((*S*)-2′-Oxo-4′-hydroxypentyl)-2-(*β*-D-glucopyranosyloxy-methyl) chromone	−6.3604	1.6246	−5.6622	0.9999	*A. vera*	[68,123]
**144**	8-Glucosyl-(2′-*O*-cinnamoyl)-7-*O*-methyl-aloediol A	−6.8459	1.3641	−6.1606	1.1397	*A. vera*	[132]
**145**	Aloeresin G	−5.6136	1.0175	−7.2021	1.2832	*A. vera*	[69,123,149]
**146**	Aloeresin H	−7.5738	1.6723	−7.5525	1.2922	*A. ferox*	[150]
**147**	Aloeresin I	−7.4396	2.2729	−7.4337	1.7717	*A. ferox*	[151]
**148**	Iso-aloesin	−6.5817	0.9104	−5.3266	1.3373	*A. vera var. chinensis*	[152]
**149**	Aloeveraside B	−7.0658	1.3476	−5.6113	1.4352	*A. vera*	[72,132]
**150**	Aloeveraside A	−7.1344	1.1636	−4.8353	1.2241	*A. vera*	[72,132]
**151**	Furoaloesone	−5.2765	1.3977	−5.3016	1.0583	*A. ferox*	[153]
III-Coumarins:							
**152**	Coumarin	−4.3565	1.3443	−3.5142	0.7049	*A. vera*	[86]
**153**	7-Demethylsiderin	−4.4857	1.2535	−4.6641	1.3044	*A. vera, A. megalacantha*	[72,86]
IV-Flavonoids:							
**154**	Apigenin	−4.8832	1.1056	−4.9431	1.4340	*A. vera*	[154]
**155**	Kaempferol	−5.5622	0.6388	−5.2731	1.3518	*A. vera*	[154]
**156**	Quercetin	−5.5045	0.8816	−5.0091	1.3859	*A. vera*	[154]
**157**	Myricetin	−4.3458	1.1650	−5.1872	1.7167	*A. vera*	[154]
**158**	Quercitrin	−5.9830	1.1253	−6.6787	1.2189	*A. vera*	[154]
**159**	Rutin	−7.7285	1.4051	−6.5175	1.7434	*A. vera*	[154]
**160**	3′,5′,6,7-Tetra-methyl ether, 5-*O*-[*α*-L-rhamnopyranosyl-(1→6)-*β*-D-galactopyranoside], 3′,4′,5,5′,6,7-Hexahydroxyflavone	−6.4680	1.4828	−7.2475	1.4030	*A. vera*	[155]
**161**	Catechin	−5.5232	0.7426	−5.0722	0.8989	*A. vera*	[154]
**162**	Epicatechin	−5.3431	1.3310	−5.0829	1.3469	*A. vera*	[154]
**163**	3′,4′,6-Tri-Me ether, 5-*O*-[*α*-L-rhamnopyranosyl-(1→6)-*β*-D-glucopyranoside], 3′,4′,5,6,7-Pentahydroxyisoflavone	0.3390	0.9914	−4.0257	1.8089	*A. vera*	[156]
V-Phenolic Compounds: a-Simple phenolic compounds:							
**164**	Pyrocatechol	−3.9037	0.5358	−3.1988	1.0732	*A. ferox*	[157]
**165**	Salicylaldehyde	−2.6754	1.0693	−3.2178	1.2797	*A. vera*	[72,86]
**166**	*p*-Cresol	−3.7780	1.1628	−3.5110	1.1472	*A. vera*	[72,86]
**167**	*p*-Hydroxyacetophenone	−4.0224	1.8629	−3.7773	0.7970	*A. ferox*	[157]
**168**	*p*-Hydroxybenzaldehyde	−3.1097	0.7939	−3.5440	1.5859	*A. ferox*	[157]
**169**	*p*-Anisaldehyde	−3.9164	1.3669	−3.4465	1.1778	*A. vera*	[72,86]
**170**	Phloretic acid	−4.3823	1.2905	−4.1107	1.0512	*A. vera*	[72,86]
**171**	Methyl-3-(4-hydroxyphenyl) propionate	−4.7496	0.8674	−3.9136	0.9518	*A. vera*	[72,86]
**172**	Coumaric acid	−4.5055	1.4107	−3.6587	1.1690	*A. vera*	[154]
**173**	Caffeic acid	−4.1969	1.2736	−3.8859	1.0162	*A. vera*	[154]
**174**	Ferulic acid	−4.3845	0.9040	−4.4689	1.0173	*A. vera*	[154]
**175**	Sinapic acid	−5.1021	1.0097	−4.2763	1.1207	*A. vera*	[154]
**176**	Orcinol	−3.9362	1.2430	−3.7654	1.4027	*A. sp.*	[69]
**177**	Gentisic acid	−3.8595	1.6010	−3.9828	1.3232	*A. vera*	[154]
**178**	Protocatechuic acid	−3.6447	1.5645	−4.1049	1.3881	*A. vera*	[154]
**179**	Vanillic acid	−4.0256	1.0470	−4.0827	0.9452	*A. vera*	[154]
**180**	Gallic acid	−4.1961	0.5757	−3.5679	0.8059	*A. vera*	[154]
**181**	Syringic acid	−4.1467	1.2874	−4.6308	1.2947	*A. vera*	[154]
**182**	1-(2,4-Dihydroxy-6-methylphenyl) ethanone	−3.8183	1.3698	−3.9336	0.9642	*A. vera*	[72]
b-Phenyl-Pyran and Phenyl-pyrone derivatives:							
**183**	Aloenin aglycone.	−4.8494	1.2878	−3.8957	1.3574	*A. vera, A. spp.*	[68,69,123,158,159]
**184**	6-(2,4-Dihydroxy-6-pentylphenyl)-4-hydroxy-2*H*-pyran-2-one	−3.1749	1.0339	−5.5428	1.0487	*A. arborescens*	[160]
**185**	6-[[2,4-Dihydroxy-6-[2-(4-hydroxyphenyl) ethenyl]-4-hydroxy-2*H*-pyran-2-one	−5.9742	1.4002	−5.8852	1.4624	*A. arborescens*	[160]
**186**	Aloenin A	−5.3723	1.3544	−4.5645	1.5586	*A. vera, A. arborescens*	[85,92,123,159]
**187**	2″-*O*-*trans*-*p*-coumaroyl-aloenin	−7.0328	1.6452	−6.1026	1.2183	*A. vera, A. nyeriensis, A. spicata*	[159]
**188**	4″,6″-Ethylidenealoenin	−6.3221	2.1206	−6.8580	1.0640	*A. arborescens, A. hijazensis*	[82]
**189**	Aloenin C	−4.0535	1.3070	−4.7701	1.7252	*A. sp.*	[123]
**190**	10-*O*-*β*-D-glucopyranosyl-aloenin	−6.1378	1.0013	−6.6976	1.3721	*A. sp., A. vera, A. spicata*	[127,136]
**191**	Aloenin B	−7.0573	1.9426	−4.7766	1.3631	*A. hijazensis, A. spicata, A. vera*	[68,69,82,123,136]
**192**	2,4-Dihydroxy-*β*-(4-hydroxyphenyl)-5-(4-methoxy-2-oxo-2*H*-pyran-6-yl)-6-methylbenzenepropanoic acid; Et ester	−6.0558	1.2415	−6.5747	1.4078	*A. vera*	[161]
**193**	6−[[3,5-Dihydroxy-2-(1-oxohexyl) phenyl] methyl]-4-hydroxy-2*H*-pyran-2-one	−5.5175	1.3328	−5.1989	1.4473	*A. arborescens*	[160]
**194**	6-[[3,5-Dihydroxy-2-[3-(4-hydroxyphenyl)-1-oxo-2-propenyl] phenyl] methyl]-4-hydroxy-2*H*-pyran-2-one	−3.9537	1.4667	−6.8047	1.2550	*A. arborescens*	[160]
**195**	3,4-Dihydro-7-hydroxy-4-(4-hydroxyphenyl)-6-(4-methoxy-2-oxo-2*H*-pyran-6-yl)-5-methyl-2*H*-1-benzopyran-2-one	−6.0022	1.3606	−6.4475	1.1932	*A. vera*	[161]
**196**	Feralolide	−5.7980	1.8453	−5.4781	1.4521	*A. arborescent, A. ferox, A. vera*	[70,82,86,162,163]
**197**	5′-*O*-Methylferalolide	−6.3808	1.3485	−5.9990	1.3806	*A. vera*	[164]
**198**	Feralolide-3′-*O*-*β*-D-Glucopyranoside	−5.6364	1.3200	−4.3041	2.3081	*A. arborescens, A. vera*	[70,165]
**199**	3,3′-Bi(3,4-dihydro-6-methoxy-2H-1-benzopyran-4-ol). 3,3′-Bi(3,4-dihydro-4-hydroxy-6-methoxy-2H-1-benzopyran)	−5.3093	1.7270	−5.2061	1.4071	*A. vera*	[166]
c-Benzofurans:							
**200**	5-Hydroxy-3-methylnaphtho[2,3-c] furan-4(*1H*)-one	−4.3383	1.1118	−4.0958	0.7359	*A. ferox*	[71]
**201**	5-Hydroxy-3-methylnaphtho[2,3-*c*] furan-4(9*H*)-one	−4.1346	1.3867	−4.0957	0.7362	*A. ferox*	[71]
**202**	Isoeleutherol	−4.7544	1.2527	−4.5232	0.7749	*A. graminicola*	[167]
**203**	Isoeleutherol glucoside	−6.0044	1.3381	−4.6325	1.4751	*A. saponaria*	[80]
**204**	8-Hydroxy-1-methylnaphtho[2,3-c] furan-4,9-dione	−5.0447	1.3119	−4.3271	0.8506	*A. ferox*	[71]
d-Naphthalin derivatives:							
**205**	Droserone	−4.5525	1.0154	−4.1450	1.1420	*A. dawei*	[66]
**206**	Droserone-5-methyl ether	−4.8881	1.1213	−4.2859	1.2524	*A. dawei*	[66]
**207**	Hydroxydroserone	−4.9712	1.0739	−3.7656	1.9742	*A. dawei*	[66]
**208**	Ancistroquinone C	−4.8894	1.4452	−4.2108	0.5425	*A. dawei*	[66]
**209**	5,8-Dihydroxy-3-methoxy-2-methyl-1,4-naphthoquinone	−5.0591	1.0203	−3.5906	0.8949	*A. dawei*	[66]
**210**	Malvone A	−4.7003	0.6291	−4.7158	1.0717	*A. dawei*	[66]
**211**	6-Hydroxy-3,5-dimethoxy-2-methyl-1,4-naphthoquinone	−4.8603	1.1151	−4.2108	1.2529	*A. dawei*	[66]
**212**	1,8-Dimethoxynepodinol	−4.8625	1.0642	−4.1478	0.9217	*A. megalacantha*	[67]
**213**	3-Hydroxy-1-(1,7-dihydroxy-3,6-dimethoxynaphthalen-2-yl) propan-1-one	−5.8504	0.7746	−5.2376	1.0515	*A. vera*	[168]
**214**	Plicataloside	−3.4118	1.4389	−5.6793	1.2349	*A. plicatilis*	[169]
**215**	Kenyaloside	−7.2910	1.6098	−4.6954	1.3254	*a Kenyan A. spp.*	[170]
**216**	Aloveroside A	−7.5802	1.8561	−6.8612	1.5133	*A. vera, A. spp.*	[69,123,171]
**217**	Isoeleutherin	−4.3217	1.3635	−3.7234	0.6554	*A. graminicola*	[167]
**218**	1-(4-Hydroxyphenyl)-6,9-dihydroxy-7-methyl-8-acetyl-1,2-dihydro-(*3H*)-naphtho[2,1-b] pyran-3-one	−3.8117	1.5671	−6.9288	0.9758	*A. ferox*	[172]
VI-Alkaloids:							
**219**	4,7-Dichloroquinoline	−1.6976	0.2458	−3.5415	1.2153	*A. hijazensis*	[82]
**220**	N, N-Dimethyl-(+)-coniine	−1.9420	0.6273	−1.7066	1.3831	*A. sabaea*	[173]
**221**	γ-Coniceine	−4.2358	0.9461	−3.6823	0.9519	*A. sp.*	[173,174]
VII-Fatty acid derivatives:							
**222**	10-Hydroxyoctadecanoic acid	−6.1427	1.4991	−4.4657	1.1518	*A. ferox*	[157]
**223**	10-Oxooctadecanoic acid	-6.3362	1.0628	−5.8079	1.1759	*A. ferox*	[157]
**224**	Methyl-26-*O*-feruloyl-oxyhexacosanate	−7.6053	1.7939	−7.2349	1.2702	*A. megalacantha*	[67]
VIII-Miscellaneous compounds:							
**225**	Nilic acid	−4.0096	0.9979	−2.3951	0.6370	*A. littoralis*	[113]
**226**	*N*-(4-Chlorobutyl) butanamide	−4.7596	0.6115	−3.9758	0.9418	*A. sabaea*	[173]
**227**	3-Furanmethanol	−3.9177	0.9518	−3.4939	0.7289	*A. arborescens*	[175]
**228**	3, 6-Dioxo-3, 3a, 6, 6 a-tetrahydropyrrolo [3, 4-c] pyrrole-1, 4-dicarboxamide	−4.1012	1.7165	−3.8036	1.0232	*A. vera*	[176]
**229**	1-(2,4-Dihydroxy-6-methylphenyl)-1-(4-hydroxyphenyl) ethane	−4.7735	2.0838	−2.0969	1.0198	*A. ferox*	[177]
**230**	Chlorogenic acid	−4.1722	1.3907	−5.6761	1.2173	*A. vera, A. arborescens*	[154]
**231**	Pluridone; (*E*)-form	−4.9712	1.2855	−3.8181	1.0980	*A. pluridens*	[178]
**232**	Feroxidin	−4.3149	1.0601	−3.9958	1.0673	*A. ferox, A. arborescens*	[69,70,72,86,123,179,180]
**233**	Feroxin A	−5.5313	1.4467	−5.2869	1.0687	*A. spp.*	[181]
**234**	Feroxin B	−6.6446	2.1091	−6.2902	1.7466	*A. spp.*	[181]
**235**	Veracylglucan A	−5.7829	1.2199	−4.7653	0.8308	*A. vera*	[182]
**236**	Veracylglucan B	−3.0836	1.0444	−1.0368	1.0780	*A. vera*	[182]
**237**	Veracylglucan C	−6.3433	2.2092	−7.2512	1.9263	*A. vera*	[182]
