# Supplementary material for: Identification of Potential SARS-CoV-2 Main Protease and Spike Protein Inhibitors from the Genus Aloe: An In Silico Study for Drug Development
Source: Molecules. 2021 Mar 21;26(6):1767. doi: 10.3390/molecules26061767 (PMC8004122; doi:10.3390/molecules26061767)
Supplement: Supplementary file 1 [file molecules-26-01767-s001.pdf]

Supplementary

Article

Identification of Potential SARS-CoV-2 Main Protease and Spike Protein Inhibitors from the Genus *Aloe*: An *In silico* Study for Drug Development

Mohamed E. Abouelela [1], Hamdy K. Assaf [1], Reda A. Abdelhamid [1], Ehab S. Elkhyat [1], Ahmed M. Sayed [2], Tomasz Oszako [3], Lassaad Belbahri [4,*], Ahmed E. El Zowalaty [5, 6,*] and Mohamed Salaheldin. A. Abdelkader [7,*]

1 Department of Pharmacognosy, Faculty of Pharmacy, Al-Azhar University, Assiut-Branch, Assiut 71524, Egypt; m_abouelela@azhar.edu.eg (M.E.A.); hamdyss200@azhar.edu.eg (H.K.A.); reda.ahmed@azhar.edu.eg (R.A.A.); elkhayat@azhar.edu.eg (E.S.E.)
2 Department of Pharmacognosy, Faculty of Pharmacy, Nahda University, Beni-Suef 62513, Egypt; Ahmed.mohamed.sayed@nub.edu.eg
3 Department of Forest Protection, Forest Research Institute, 05-090 Sekocin Stary, Poland; T.Oszako@ibles.waw.pl
4 Laboratory of Soil Biology, University of Neuchatel, 2000 Neuchatel, Switzerland
5 Sahlgrenska Center for Cancer Research, Department of Surgery, Institute of Clinical Sciences, University of Gothenburg, 405 30 Gothenburg, Sweden
6 Wallenberg Centre for Molecular and Translational Medicine, University of Gothenburg, 405 30 Gothenburg, Sweden
7 Department of Pharmacognosy, Faculty of Pharmacy, Sohag University, Nasr City, 82524, Sohag, Egypt

* Correspondence: lassaad.belbahri@unine.ch (L.B.); ahmed.el.zowalaty@gu.se (A.E.E.Z.); m.salaheldin@pharm.sohag.edu.eg (M.S.A.A.)

Citation: Abouelela M. E., Assaf H. K, Abdelhamid R. A., Elkhyat E. S., Sayed A. M, Oszako T., Belbahri L., El Zowalaty A. E. and Abdelkader M. S. Identification of Potential SARS-CoV-2 Main Protease and Spike Protein Inhibitors from *Aloe* genus: *In silico* study for drug development. *Molecules* **2021**, *26*, 1767. https://doi.org/10.3390/10.3390/molecules26061767

Academic Editors: Shugeng Cao, Mostafa Rateb and Jianguang Luo
Received: 24 February 2021
Accepted: 18 March 2021
Published: 21 March 2021

Publisher's Note: MDPI stays neutral with regard to jurisdictional claims in published maps and institutional affiliations.

Copyright: © 2021 by the authors. Licensee MDPI, Basel, Switzerland. This article is an open access article distributed under the terms and conditions of the Creative Commons Attribution (CC BY) license (http://creativecommons.org/licenses/by/4.0/).

Page	Contents
3	**Figure S1**: Compounds isolated from genus Aloe (**1-17**)
4	**Figure S2**: Compounds isolated from genus Aloe (**18-31**)
5	**Figure S3**: Compounds isolated from genus Aloe (**32-47**)
6	**Figure S4**: Compounds isolated from genus Aloe (**48-60**)
7	**Figure S5**: Compounds isolated from genus Aloe (**61-75**)
8	**Figure S6**: Compounds isolated from genus Aloe (**76-92**)
9	**Figure S7**: Compounds isolated from genus Aloe (**93-107**)
10	**Figure S8**: Compounds isolated from genus Aloe (**108-118**)
11	**Figure S9**: Compounds isolated from genus Aloe (**119-127**)
12	**Figure S10**: Compounds isolated from genus Aloe (**128-136**)
13	**Figure S11**: Compounds isolated from genus Aloe (**137-144**)
14	**Figure S12**: Compounds isolated from genus Aloe (**145-153**)
15	**Figure S13**: Compounds isolated from genus Aloe (**154-168**)
16	**Figure S14**: Compounds isolated from genus Aloe (**169-182**)
17	**Figure S15**: Compounds isolated from genus Aloe (**183-194**)
18	**Figure S16**: Compounds isolated from genus Aloe (**195-211**)
19	**Figure S17**: Compounds isolated from genus Aloe (**212-231**)
20	**Figure S18**: Compounds isolated from genus Aloe (**232-237**)
21	**Table S1.** Predicted pharmacokinetics of top scoring compounds.

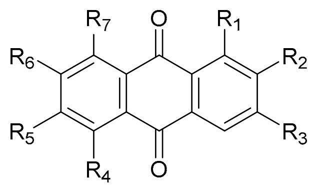

	R_1	R_2	R_3	R_4	R_5	R_6	R_7
**1**	OH	H	CH_3	H	H	H	OH
**2**	OH	H	CH_3	OH	H	H	OCH_3
**3**	OH	H	$CH_2(OH)$	H	H	H	OH
**4**	OH	H	$CH_2(OH)$	H	H	OH	OH
**5**	OH	H	CH_3	H	H	OH	OH
**6**	OH	H	CH_3	H	H	OH	OCH_3
**7**	OH	H	CH_3	H	OH	H	OH
**8**	CH_3	H	OH	H	H	H	OH
**9**	CH_3	$COOCH_3$	OH	H	H	H	OH
**10**	CH_3	$COOCH_3$	OH	H	H	H	OCH_3
**11**	OH	H	CH_3	OH	H	H	OH
**12**	OH	H	CH_3	OCH_3	H	H	H
**13**	OH	H	CH_3	OH	H	OCH_3	OH
**14**	CH_3	H	OH	H	OH	H	OH
**15**	CH_3	$COOCH_3$	OH	H	OH	H	OH
**16**	OH	H	CH_3	H	*O*-Prenyl	H	OH
**17**	OH	H	CH_3	H	*O*-Prenyl-Prenyl	H	OH

Figure S1. Compounds isolated from genus Aloe (**1-17**).

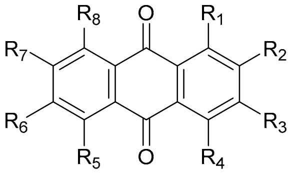

	R_1	R_2	R_3	R_4	R_5	R_6	R_7	R_8
**18**	OH	NO_2	$CH_2(OH)$	NO_2	NO_2	H	NO_2	OH
**19**	OH	H	CH_3	H	H	H	*O*-*β*-D-Glc.	OH
**20**	OH	H	CH_2-*O*-Rha.	H	H	H	H	OH

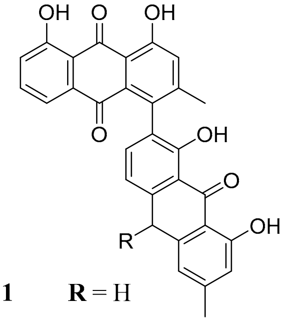

21 **R** = H

22 **R** = CO

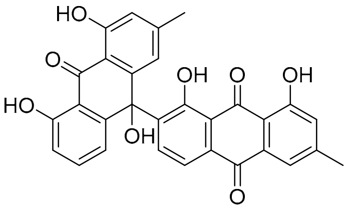

23

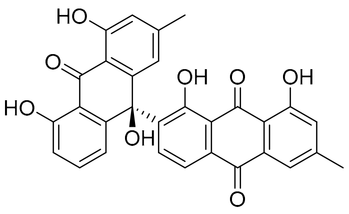

24

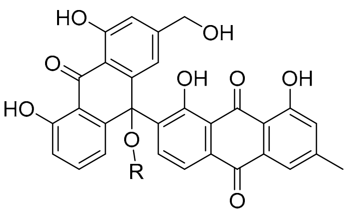

25 **R** = H

26 **R** = CH_3

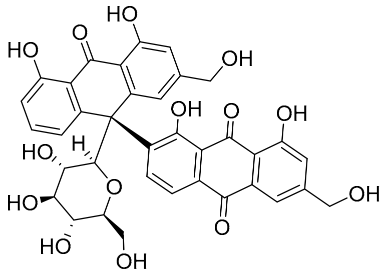

27

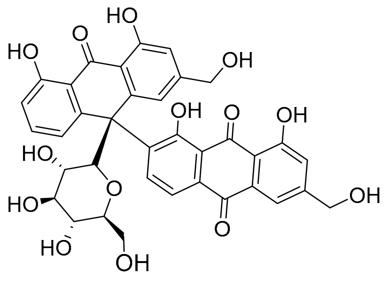

28

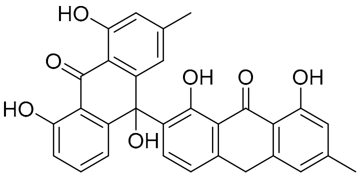

29

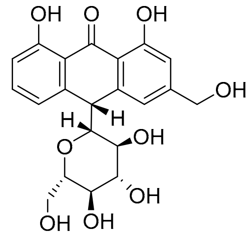

30

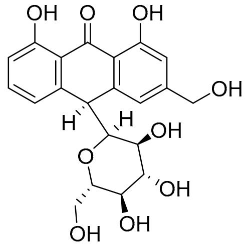

31

Figure S2: Compounds isolated from genus Aloe (**18-31**)

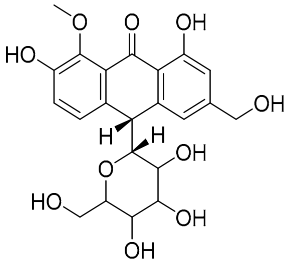

32

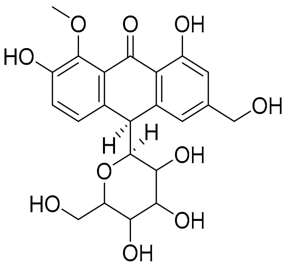

33

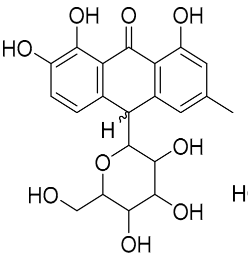

34

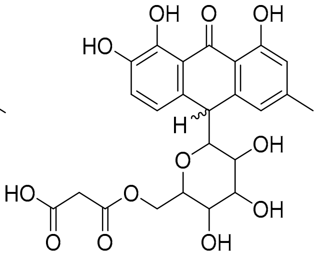

35

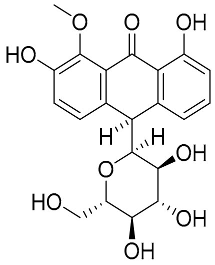

36

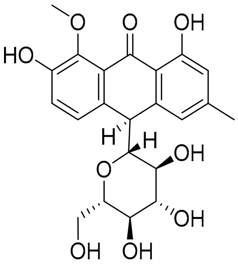

37

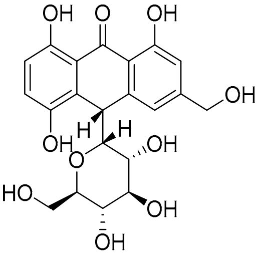

38

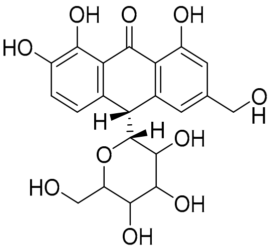

39

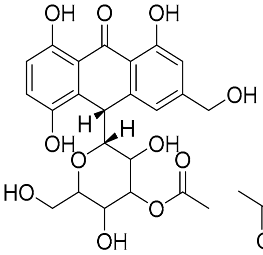

40

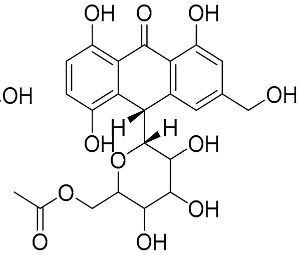

41

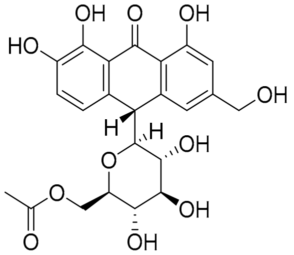

42

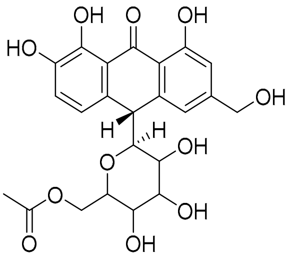

43

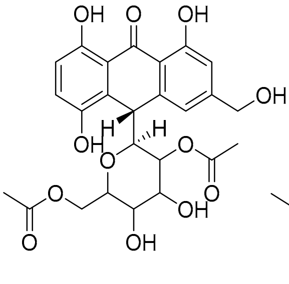

44

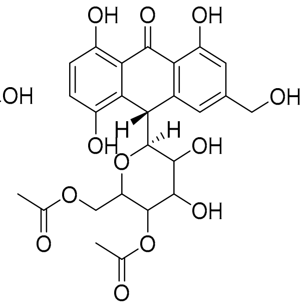

45

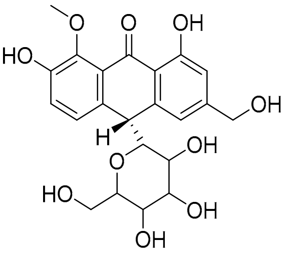

46

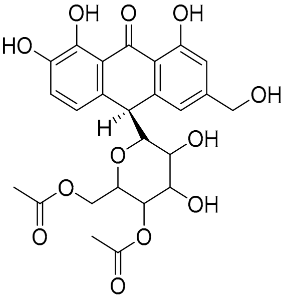

47

Figure S3: Compounds isolated from genus Aloe (**32-47**)

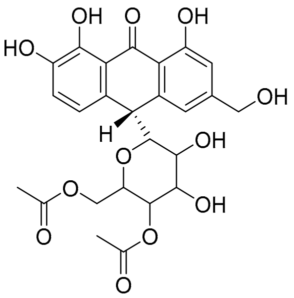

48

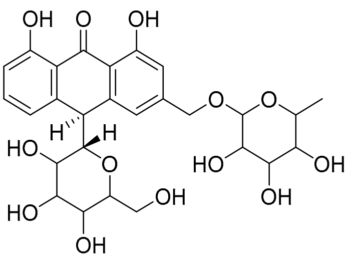

49

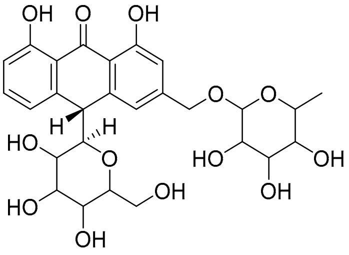

50

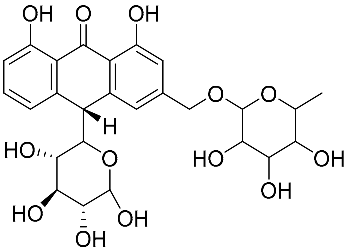

51

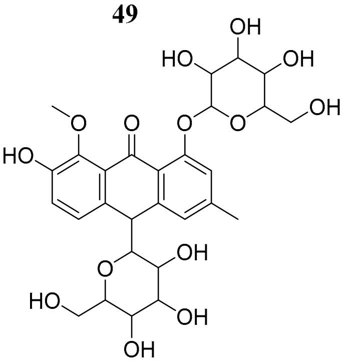

52

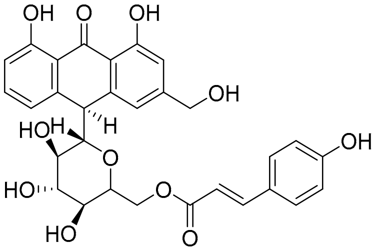

53

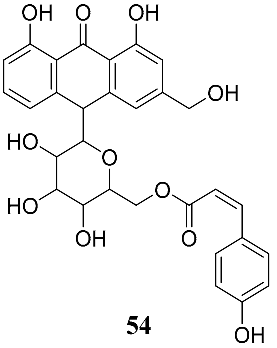

54

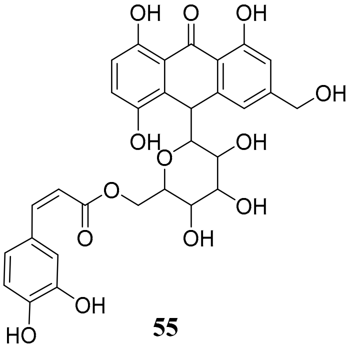

55

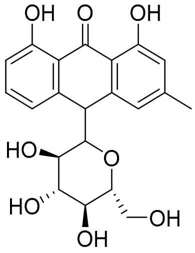

56

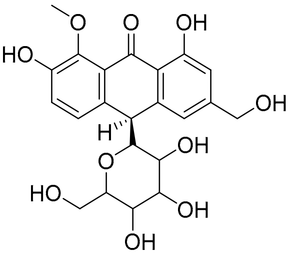

57

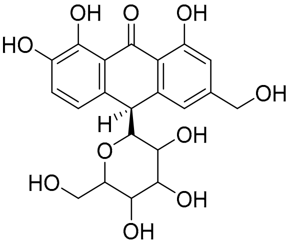

58

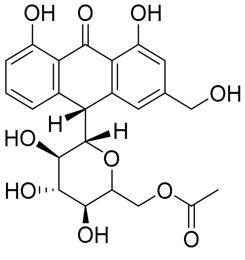

59

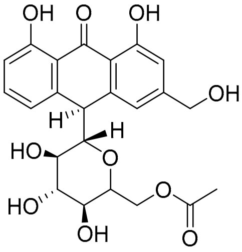

60

Figure S4: Compounds isolated from genus Aloe (**48-60**)

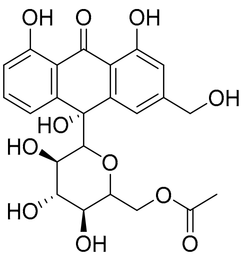
61

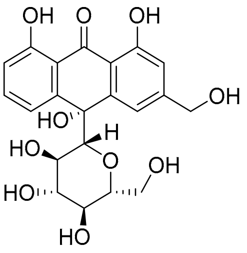
62

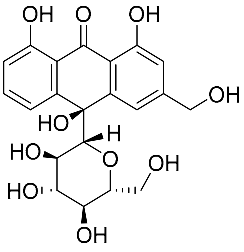
63

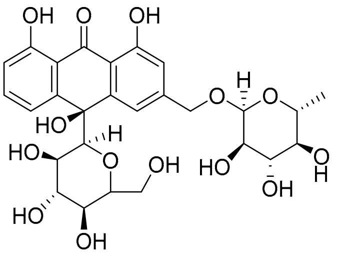
64

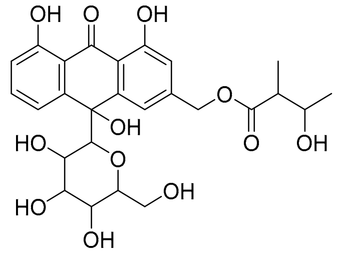
65

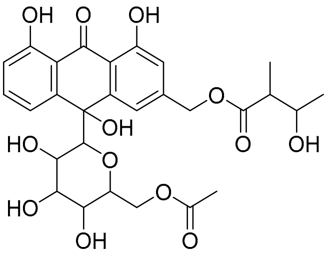
66

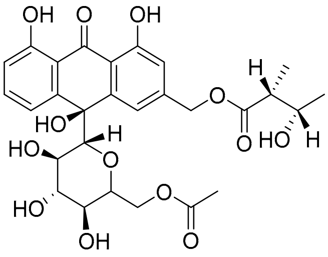
67

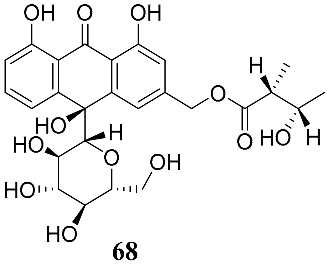
68

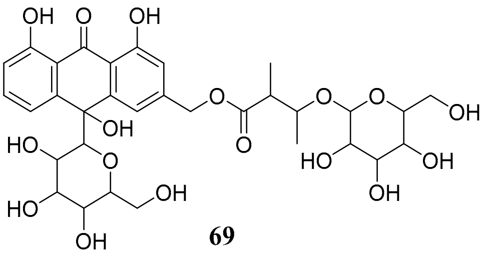
69

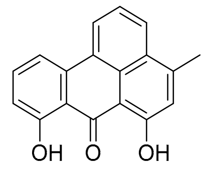
70

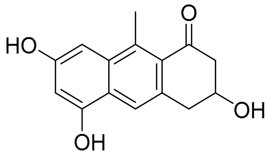
71

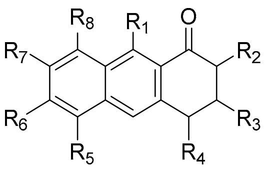

	R_1	R_2	R_3	R_4	R_5	R_6	R_7	R_8
**72**	CH_3	H	OH	H	OH	H	OH	H
**73**	OH	H	OH	H	H	OH	H	CH_3
**74**	OH	H	OH	H	H	OCH_3	H	CH_3
**75**	OH	OCH_3	H	OH	H	CH_3	H	OH

Figure S5: Compounds isolated from genus Aloe (**61-75**)

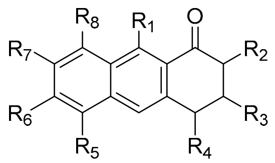

	R_1	R_2	R_3	R_4	R_5	R_6	R_7	R_8
**76**	OH	H	OH	H	H	OH	$COOCH_3$	CH_3
**77**	OH	H	H	OH	H	CH_3	H	OH
**78**	OH	H	H	OH	H	CH_3	H	OCH_3
**79**	OH	H	H	OH	H	CH_3	H	*O*-*β*-D-Glc.
**80**	OH	OCH_3	H	OH	H	CH_3	H	*O*-*β*-D-Glc.
**81**	OH	OH	H	*O*-*β*-D-Glc.	H	CH_3	H	OH
**82**	OH	OCH_3	H	*O*-*β*-D-Glc.	H	CH_3	H	OH
**83**	OH	H	OH	H	H	*O*-*β*-D-Glc.	H	CH_3
**84**	OH	H	*O*-*β*-D-Glc.	H	H	OH	$COOCH_3$	CH_3

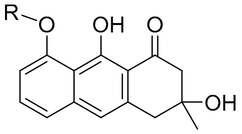

85 **R** = H

86 **R** = CH_3

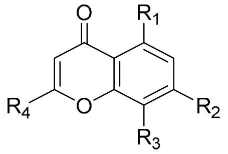

	R_1	R_2	R_3	R_4
**87**	CH_3	OH	H	OH
**88**	CH_3	OH	H	CH_3
**89**	$CH_2(OH)$	OH	H	CH_3
**90**	$CH_2(OH)$	OCH_3	H	CH_3
**91**	CH_3	OH	H	CH_2COCH_3
**92**	CH_3	OH	H	$CH_2CH(OH)CH_3$

Figure S6: Compounds isolated from genus Aloe (**76-92**)

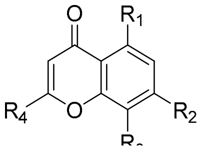

	R_1	R_2	R_3	R_4
**93**	OH	OCH_3	H	$CH_2(OH)$
**94**	OH	OH	H	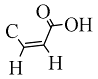
**95**	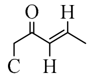	H	H	$CH_2(OH)$
**96**	CH_3	OH	$CH_2COCH_2(OH)$	CH_2COCH_3
**97**	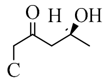	H	H	$CH_2(OH)$
**98**	CH_3	OCH_3	β-D-Glc.	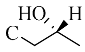
**99**	CH_3	OCH_3	β-D-Glc.	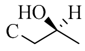
**100**	CH_3	OH	β-D-Glc.	$CH_2CH(OH)CH_3$
**101**	CH_3	OCH_3	β-D-Glc.	$CH_2(OH)CH(OH)CH_3$
**102**	CH_3	OCH_3	β-D-Glc.	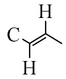
**103**	CH_3	OH	β-D-Glc.	CH_3
**104**	OH	OH	β-D-Glc.	CH_3
**105**	OH	OH	α-D-Glc.	CH_3
**106**	CH_3	OH	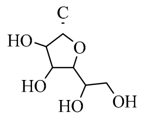	CH_2COCH_3
**107**	CH_3	OH	β-D-Glc.	CH_2COCH_3

Figure S7: Compounds isolated from genus Aloe (**93-107**)

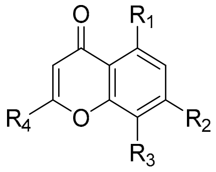

	R_1	R_2	R_3	R_5
**108**	CH_3	OCH_3	*β*-D-Glc.	CH_2COCH_3
**109**	CH_3	OH	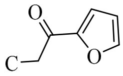	CH_2COCH_3
**110**	CH_3	OH	*β*-D-Glc.	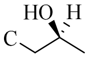
**111**	CH_3	OH	*β*-D-Glc.	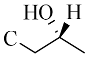
**112**	CH_3	OCH_3	*β*-D-Glc.	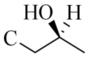
**113**	CH_3	OCH_3	*β*-D-Glc.	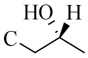
**114**	CH_3	OH	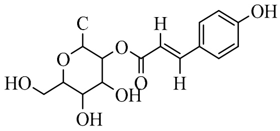	$CH_2CH(OH)CH_3$
**115**	CH_3	OH	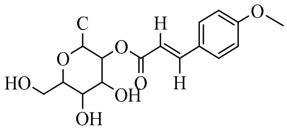	$CH_2CH(OH)CH_3$
**116**	CH_3	OCH_3	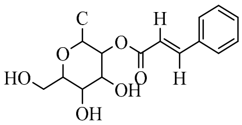	$CH(OH)CH(OH)CH_3$
**117**	CH_3	OCH_3	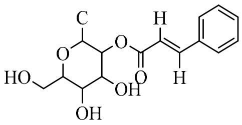	$CH_2CH(OH)CH_3$
**118**	CH_3	OCH_3	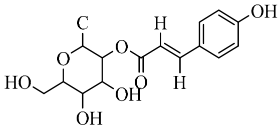	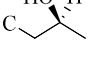

Figure S8: Compounds isolated from genus Aloe (**108-118**)

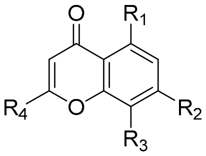

	R_1	R_2	R_3	R_4
**119**	CH_3	OCH_3	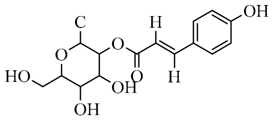	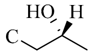
**120**	CH_3	OCH_3	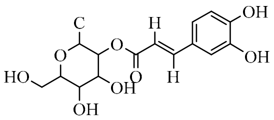	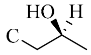
**121**	CH_3	OCH_3	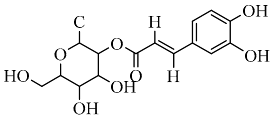	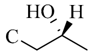
**122**	CH_3	OCH_3	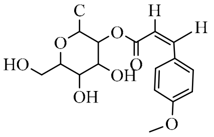	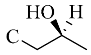
**123**	CH_3	OCH_3	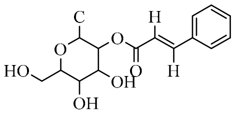	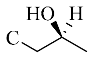
**124**	CH_3	OCH_3	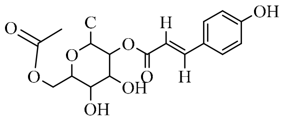	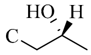
**125**	CH_3	OH	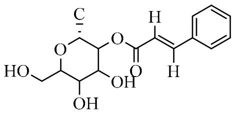	CH_2COCH_3
**126**	CH_3	OCH_3	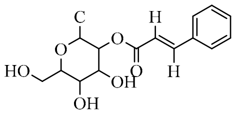	CH_2COCH_3
**127**	CH_3	OCH_3	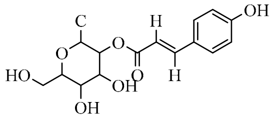	CH_2COCH_3

Figure S9: Compounds isolated from genus Aloe (**119-127**)

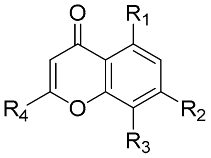

	R_1	R_2	R_3	R_4
**128**	CH_3	OH	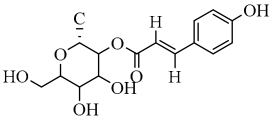	CH_2COCH_3
**129**	CH_3	OH	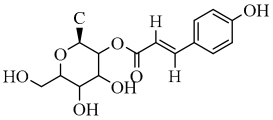	CH_2COCH_3
**130**	CH_3	OH	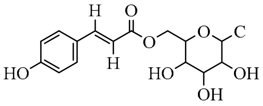	CH_2COCH_3
**131**	CH_3	OCH_3	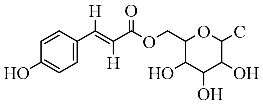	CH_2COCH_3
**132**	CH_3	OCH_3	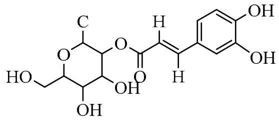	CH_2COCH_3
**133**	CH_3	OH	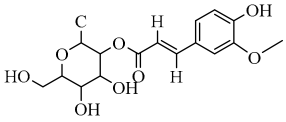	CH_2COCH_3
**134**	CH_3	OCH_3	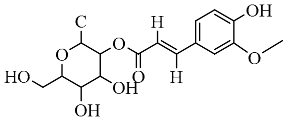	CH_2COCH_3
**135**	CH_3	OH	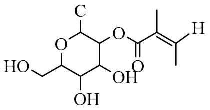	CH_2COCH_3
**136**	CH_3	OH	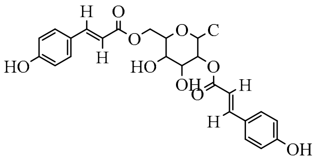	CH_2COCH_3

Figure S10: Compounds isolated from genus Aloe (**128-136**)

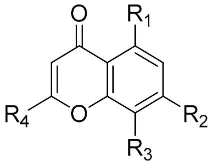

	R_1	R_2	R_3	R_4
**137**	CH_3	*O*-*β*-D-Glc.	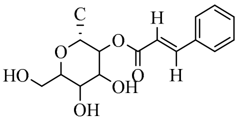	CH_2COCH_3
**138**	CH_3	*O*-*β*-D-Glc.	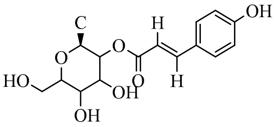	CH_2COCH_3
**139**	CH_3	OCH_3	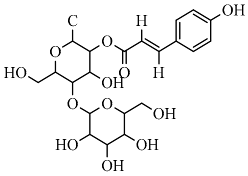	$CH_2CH(OH)CH_3$
**140**	CH_3	OCH_3	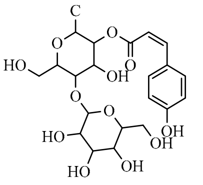	$CH_2CH(OH)CH_3$
**141**	CH_3	OH	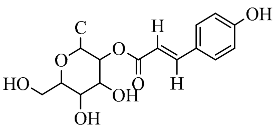	COOH
**142**	CH_3	OCH_3	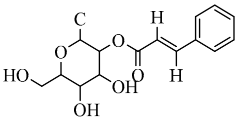	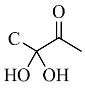
**143**	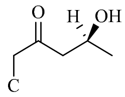	H	H	CH_2*O*-*β*-D-Glc.
**144**	CH_3	OCH_3	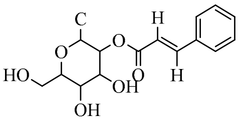	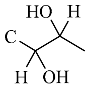

Figure S11: Compounds isolated from genus Aloe (**137-144**)

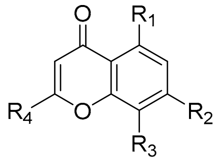

	R_1	R_2	R_3	R_4
**145**	CH_3	OCH_3	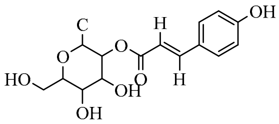	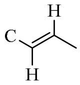
**146**	CH_3	OH	*β*-D-Glc.	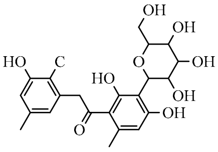
**147**	CH_3	OH	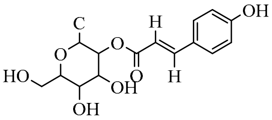	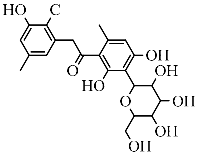

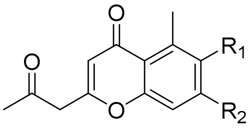

**148**	R_1 = *β*-D-Glc.	R_2 = H
**149**	R_1 = 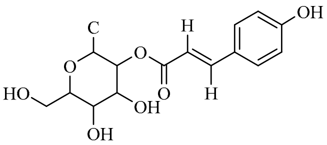	R_2 = H
**150**	R_1 = 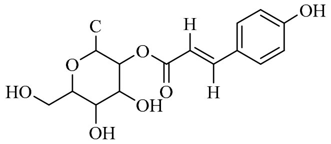	R_2 = CH_3

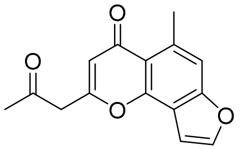

151

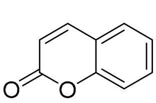

152

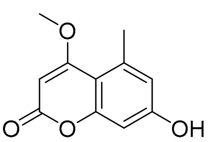

153

Figure S12: Compounds isolated from genus Aloe (**145-153**)

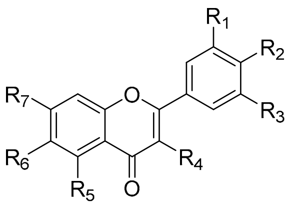

	R_1	R_2	R_3	R_4	R_5	R_6	R_7
**154**	H	OH	H	H	OH	H	OH
**155**	H	OH	H	OH	OH	H	OH
**156**	H	OH	OH	OH	OH	H	OH
**157**	OH	OH	OH	OH	OH	H	OH
**158**	OH	OH	H	*O*-*α*-L-Rha.	OH	H	OH
**159**	OH	OH	H	*O*-*β*-D-Glc.-6-*O*-*α*-L-Rha.	OH	H	OH
**160**	OCH_3	OH	OCH_3	*O*-*β*-D-Glc.-6-*O*-*α*-L-Rha.	OH	OCH_3	OCH_3

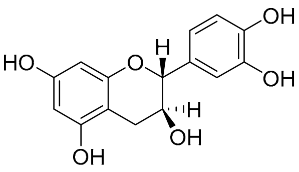

161

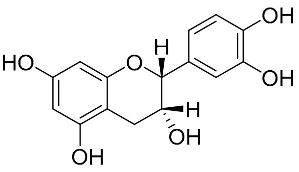

162

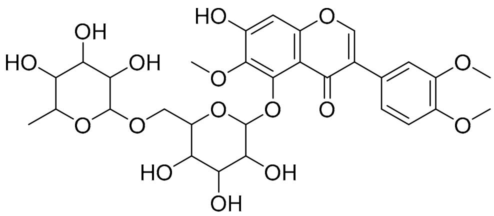

163

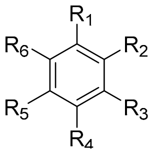

	R_1	R_2	R_3	R_4	R_5	R_6
**164**	OH	OH	H	H	H	H
**165**	OH	CHO	H	H	H	H
**166**	CH_3	H	H	OH	H	H
**167**	$COCH_3$	H	H	OH	H	H
**168**	CHO	H	H	OH	H	H

Figure S13: Compounds isolated from genus Aloe (**154-168**)

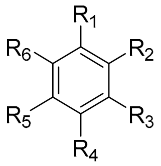

	R_1	R_2	R_3	R_4	R_5	R_6
**169**	CHO	H	H	OCH_3	H	H
**170**	CH_2CH_2CO OH	H	H	OH	H	H
**171**	CH_2CH_2CO OCH_3	H	H	OH	H	H
**172**	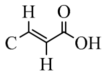	H	H	OH	H	H
**173**	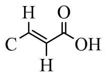	H	H	OH	OH	H
**174**	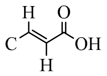	H	H	OH	OCH_3	H
**175**	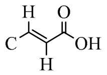	H	OCH_3	OH	OCH_3	H
**176**	OH	H	CH_3	H	OH	H
**177**	COOH	OH	H	H	OH	H
**178**	COOH	H	H	OH	OH	H
**179**	COOH	H	H	OH	OCH_3	H
**180**	COOH	H	OH	OH	OH	H
**181**	COOH	H	OCH_3	OH	OCH_3	H
**182**	$COCH_3$	CH_3	H	OH	H	OH

Figure S14: Compounds isolated from genus Aloe (**169-182**)

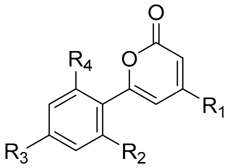

	R_1	R_2	R_3	R_4
**183**	OCH_3	OH	OH	CH_3
**184**	OH	OH	OH	Pentyl
**185**	OH	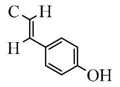	OH	OH
**186**	OCH_3	CH_3	OH	*O*-*β*-D-Glc.
**187**	OCH_3	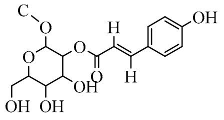	OH	CH_3
**188**	OCH_3	CH_3	OH	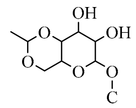
**189**	CH_3	CH_3	*O*-*β*-D-Glc.	*O*-*β*-D-Glc.
**190**	OCH_3	CH_3	*O*-*β*-D-Glc.	*O*-*β*-D-Glc.
**191**	OCH_3	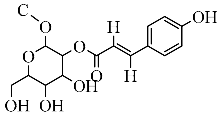	*O*-*β*-D-Glc.	CH_3

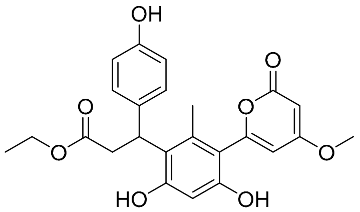

192

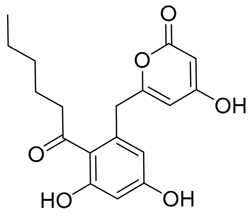

193

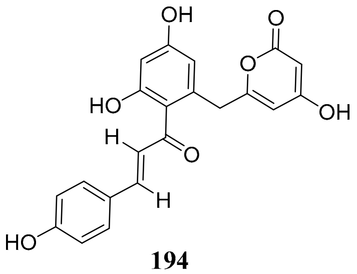

194

Figure S15: Compounds isolated from genus Aloe (**183-194**)

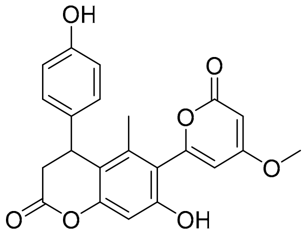

195

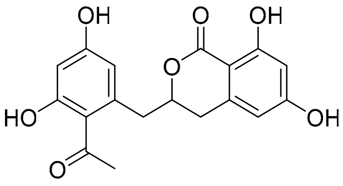

196

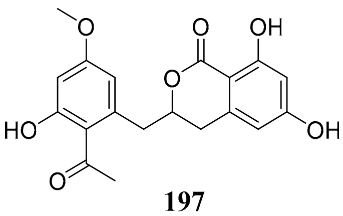

197

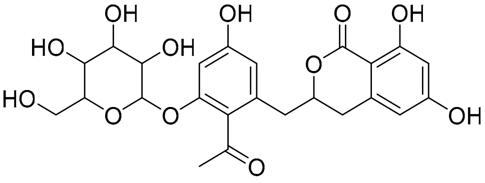

198

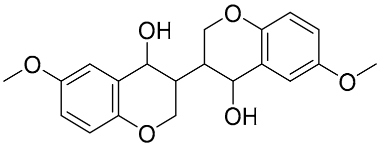

199

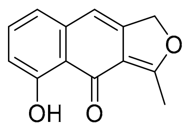

200

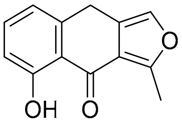

201

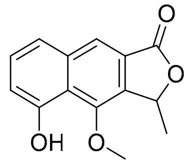

202

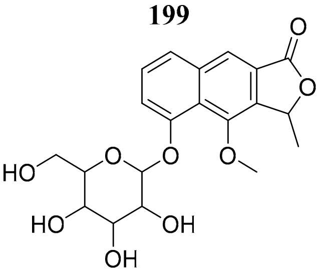

203

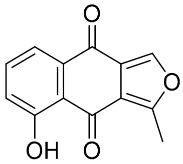

204

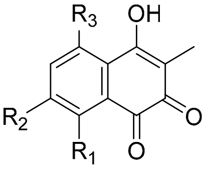

	**R_1**	**R_2**	**R_3**
**205**	OH	H	H
**206**	OCH_3	H	H
**207**	OH	H	OH
**208**	OCH_3	OCH_3	H

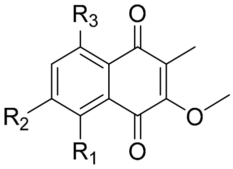

	**R_1**	**R_2**	**R_3**
**209**	OH	H	OH
**210**	OH	OH	H
**211**	OCH_3	OH	H

Figure S16: Compounds isolated from genus Aloe (**195-211**)

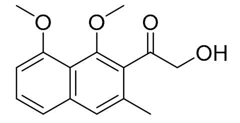

212

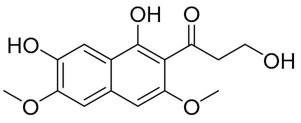

213

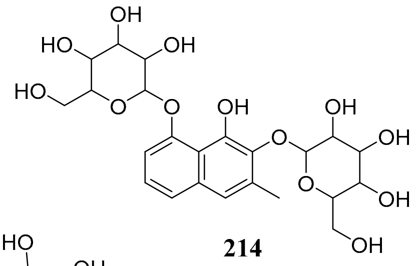

214

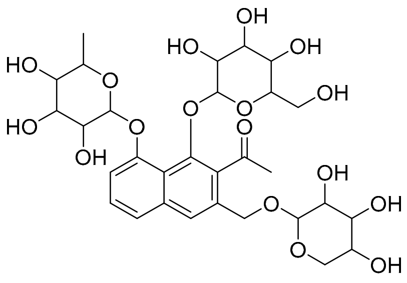

215

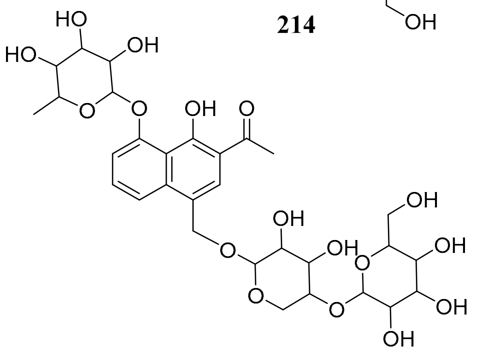

216

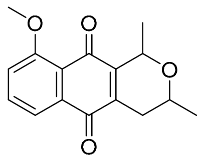

217

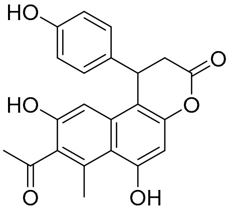

218

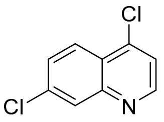

219

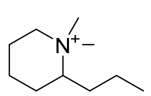

220

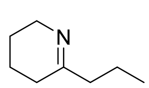

221

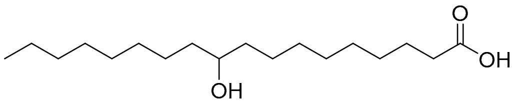

222

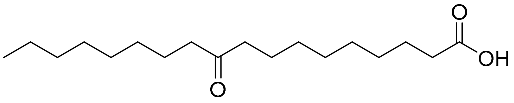

223

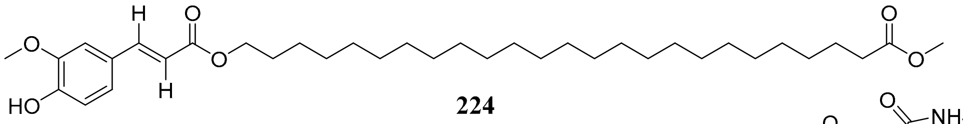

224

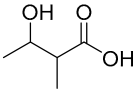

225

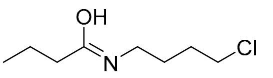

226

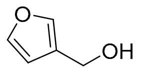

227

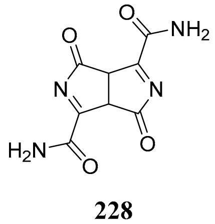

228

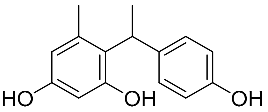

229

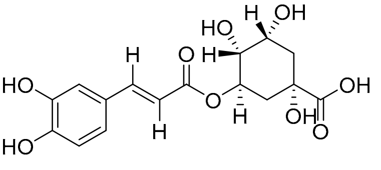

230

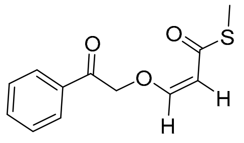

231

Figure S17: Compounds isolated from genus Aloe (**212**-**231**)

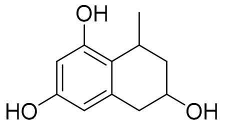

232

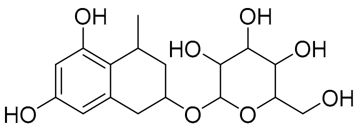

233

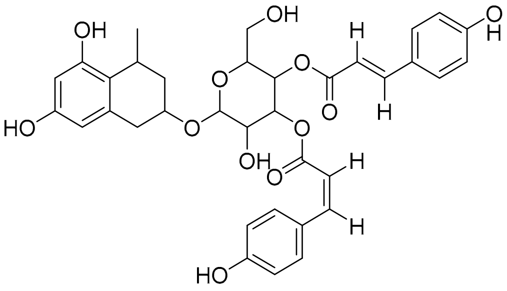

234

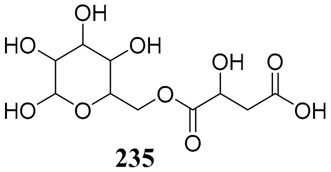

235

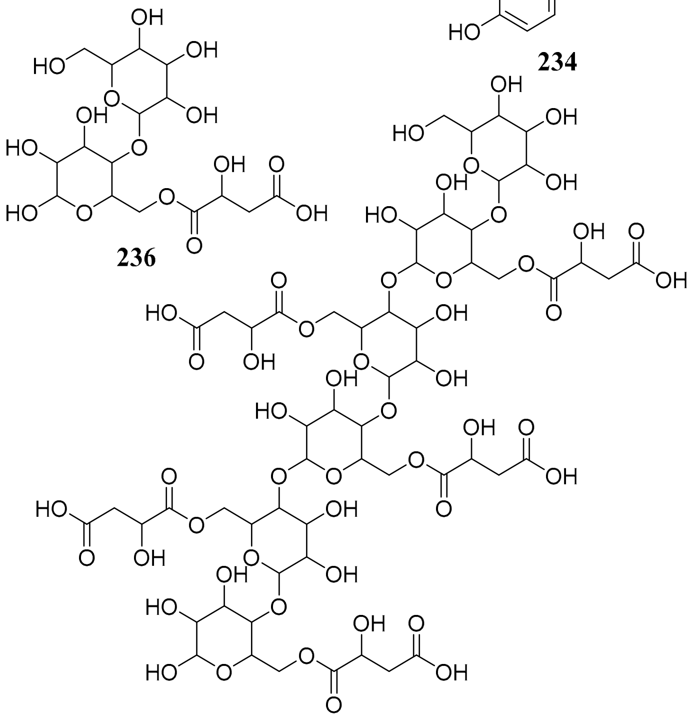

236

237

Figure S18: Compounds isolated from genus Aloe (**232**-**237**)

Table 1. Predicted pharmacokinetics of top scoring compounds.

Compound No.	115	120	131	132	134	159
**BBB**	0.044	0.041	0.0215873	0.03	0.02	0.02
**Buffer_solubility_mg_L**	12.77	18.077	4.84474	10.88	4.19	28.88
**Caco2**	12.86	10.71	11.4372	10.62	9.20	7.91
**CYP_2C19_inhibition**	Inhibitor	Inhibitor	Inhibitor	Inhibitor	Inhibitor	Inhibitor
**CYP_2C9_inhibition**	Inhibitor	Inhibitor	Inhibitor	Inhibitor	Inhibitor	Inhibitor
**CYP_2D6_inhibition**	Non	Non	Non	Non	Non	Non
**CYP_2D6_substrate**	Non	Non	Non	Non	Non	Non
**CYP_3A4_inhibition**	Inhibitor	Inhibitor	Inhibitor	Inhibitor	Inhibitor	Inhibitor
**CYP_3A4_substrate**	Substrate	Weakly	Weakly	Weakly	Weakly	Weakly
**HIA**	77.11	57.61	82.803611	66.18	79.98	2.86
**MDCK**	0.051	0.05	0.0511802	0.053	0.05	0.32
**Pgp_inhibition**	Inhibitor	Non	Non	Non	Non	Non
**Plasma_Protein_Binding**	77.20	73.52	74.833673	68.96	68.79	43.89
**Pure_water_solubility_mg_L**	3.29	4.86	3.74378	4.05	1.88	217.20
**Skin_Permeability**	-3.73	-3.85	-3.65856	-3.80	-3.65	-4.66
**SKlogD_value**	1.72	1.58	1.44	1.49	1.63	-1.11
**SKlogP_value**	1.72	1.58	1.44	1.49	1.63	-1.11
**SKlogS_buffer**	-4.63	-4.50	-5.058	-4.72	-5.14	-4.32
**SKlogS_pure**	-5.22	-5.07	-5.17	-5.14	-5.49	-3.44
